# Electron Modulation and Morphology Engineering Jointly Accelerate Oxygen Reaction to Enhance Zn‐Air Battery Performance

**DOI:** 10.1002/advs.202205889

**Published:** 2023-01-22

**Authors:** Xue Zhao, Jianbing Chen, Zenghui Bi, Songqing Chen, Ligang Feng, Xiaohai Zhou, Haibo Zhang, Yingtang Zhou, Thomas Wågberg, Guangzhi Hu

**Affiliations:** ^1^ National Engineering Research Center for Marine Aquaculture Marine Science and Technology College Zhejiang Ocean University Zhoushan 316004 China; ^2^ Research Academy of Non‐metallic Mining Industry Development Materials and Environmental Engineering College Chizhou University Chizhou 247000 China; ^3^ Institute for Ecological Research and Pollution Control of Plateau Lakes School of Ecology and Environmental Science Yunnan University Kunming 650504 China; ^4^ School of Chemistry and Chemical Engineering Yangzhou University Yangzhou 225009 China; ^5^ College of Chemistry and Molecular Sciences Wuhan University Wuhan 430072 China; ^6^ Department of Physics Umeå University Umeå S‐901 87 Sweden

**Keywords:** electron modulation, heteronuclear Fe—Co biatomic, morphology engineering, oxygen reaction, Zn‐air batteries

## Abstract

Combining morphological control engineering and diatomic coupling strategies, heteronuclear Fe—Co bimetals are efficiently intercalated into nitrogen‐doped carbon materials with star‐like to simultaneously accelerate oxygen reduction reaction (ORR) and oxygen evolution reaction (OER). The half‐wave potential and kinetic current density of the ORR driven by FeCoNC/SL surpass the commercial Pt/C catalyst. The overpotential of OER is as low as 316 mV (*η*_10_), and the mass activity is at least 3.2 and 9.4 times that of mononuclear CoNC/SL and FeNC/SL, respectively. The power density and specific capacity of the Zn‐air battery with FeCoNC/SL as air cathode are as high as 224.8 mW cm^−2^ and 803 mAh g^−1^, respectively. Morphologically, FeCoNC/SL endows more reactive sites and accelerates the process of oxygen reaction. Density functional theory reveals the active site of the heteronuclear diatomic, and the formation of FeCoN5C configuration can effectively tune the d‐band center and electronic structure. The redistribution of electrons provides conditions for fast electron exchange, and the change of the center of the d‐band avoids the strong adsorption of intermediate species to simultaneously take into account both ORR and OER and thus achieve high‐performance Zn‐air batteries.

## Graphical abstract

Morphology‐controlled engineering combined with diatomic coupling strategy to induce heteronuclear Fe—Co bimetallic with differentiated electron distribution accelerates oxygen reaction for efficient operation of zinc‐air battery energy storage devices.

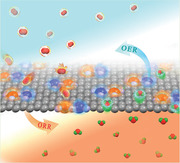

## 1 Introduction

Nearly 64.5% of the world's electricity supply comes from fossil fuel, which situation should be changed today when nonrenewable resources are continuously depleted and environmental problems are prominent.^[^ ^1^, ^2^ ^]^ Nowadays, most countries have turned their attention to the generation of clean energy, such as solar energy, wind energy, water (potential) energy, and nuclear energy. In this context, the development of a new generation of electric energy storage and conversion equipment is of great significance to the storage of clean energy and the application of different scenarios.^[^ ^3^ ^]^ Although lithium‐ion batteries have achieved great success in commercialization, they still have to face problems such as low energy density (less than 350 W h kg_Zn_^−1^) and potential safety hazards.^[^ ^1^, ^4^ ^]^ Different from lithium, sodium, potassium, magnesium, and aluminum‐air batteries, zinc‐air batteries have the advantages of high safety, low cost, higher energy density (about 1086 W h kg_Zn_^−1^) and discharge voltage (about 1.65 V), so it has become an ideal energy storage and conversion equipment.^[^ ^1^, ^5^ ^]^ At present, the performance of zinc‐air batteries (ZAB) is still limited by the slow oxygen reduction reaction (ORR) and oxygen evolution reaction (OER),^[^ ^6^ ^]^ so the development of efficient ORR and OER catalysts is the priority.

As we all know, so far Pt/C materials are still the best catalyst for driving ORR and have been commercialized.^[^ ^7^ ^]^ However, the problems of Pt/C catalysts also affect its widespread use, such as high cost (scarce resources), poor electrochemical stability, and easy CO poisoning.^[^ ^8^ ^]^ In this context, researchers have to consider other potential ORR catalysts to achieve a balance between performance and cost. Fortunately, it has been inspired by biological porphyrin catalysts that single‐atom materials with M‐N‐C configuration are conducive to achieving moderate O_2_ adsorption (formation of M—O bonds), such as heme‐like Fe‐N‐C materials.^[^ ^9^ ^]^ Many ORR catalysts with M‐N‐C configuration have been developed and effective ORR has been achieved, including Fe‐N‐C,^[^ ^10^ ^]^ Co‐N‐C,^[^ ^11^, ^12^ ^]^ Zn‐N‐C,^[^ ^12^ ^]^ Cu‐N‐C,^[^ ^13^, ^14^ ^]^ Se‐N‐C,^[^ ^15^ ^]^ etc., these achievements have shaken the status of Pt/C catalysts to a certain extent. Even so, there is still a long way to go to design non‐noble metal single‐atom materials with ORR activities surpassing those of Pt/C catalyst. On the other hand, although the catalysts with OER activity are not Pt‐based materials, the catalysts exhibiting excellent activity are Ru‐ and Ir‐based materials, such as RuO_2_ and IrO_2_. The use of these noble metal compounds (or complexes) will also increase the cost burden, so it is also necessary to find ways to reduce costs while ensuring catalytic activity. Currently, many studies are focusing on the development of non‐noble metal‐based materials with OER activity, mainly Ni, Co, Fe, Mn and their oxides, carbides, or hydroxides.^[^ ^16^ ^]^ It has been reported that the OER activity of some non‐noble metal‐based catalysts has surpassed that of RuO_2_ or IrO_2_, such as Co/N‐CNT,^[^ ^17^ ^]^ Fe/Fe_3_C/MC,^[^ ^18^ ^]^ Co‐CeO_2_/C,^[^ ^19^ ^]^ and CoFe_2_O_4_.^[^ ^20^ ^]^ Non‐noble metal‐based catalysts have shown attractive potential in catalyzing OER, but they also face urgent problems to be solved. Among them, stability is the biggest obstacle restricting non‐noble metals and their oxides, carbides, and hydroxides as OER catalysts,^[^ ^21^ ^]^ whose physical and chemical properties will be changed in the redox reaction and thus lose OER activity. In this case, it is necessary to explore reasonable ways to improve the electrochemical stability of non‐noble metal catalysts, and the formation of a single‐atom state in a coordinated form may be the best way. For example, after the formation of FeN_4_, the central Fe atom will combine with N in the form of coordination bonds, so it can resist the destruction of oxidation or reduction potential. However, the OER activity of single‐atom catalysts seems to be at a low value, and the performance is far behind Ru‐ and Ir‐based materials, and some Co‐, Fe‐, and Ni‐containing oxides and hydroxides. Therefore, it is necessary to further regulate the state of the single‐atom central metal to stimulate its OER activity.

The performance of catalysts in terms of ORR and OER can be improved to a certain extent by adjusting the electronic structure of the metal site in the single atom or controlling the morphology of the single‐atom material.^[^ ^22^ ^]^ Among them, the formation of a bimetallic atom structure is more conducive to the splitting of O_2_, because the difference in electronegativity between different metals is conducive to the transfer of electrons.^[^ ^23^ ^]^ Jiang et al. reported that Co_2_@C_2_N has a higher adsorption energy for O_2_ than Co@C_2_N, the former being able to elongate the O—O of O_2_ to 1.47 Å, while the latter was only able to elongate to 1.33 Å.^[^ ^24^ ^]^ Therefore, Co_2_@C_2_N has better ORR activity than Co@C_2_N, which is attributed to the shift of the d‐band center after diatom formation. In the report of Sun et al., the spin polarization of Fe—Co diatoms can promote the parallel spin alignment of O_2_ molecules, thus promoting the activation of O_2_ and the formation of O—O bonds in OER.^[^ ^25^ ^]^ Another report also showed that the formation of Fe—Co diatoms can enhance the ORR and OER activities, in which the activity of Co is higher than that of Fe, because the presence of Fe can change the electronic structure of Co, thereby improving the bonding behavior of *O intermediates.^[^ ^26^ ^]^ These phenomena suggest that the introduction of another metal atom in the single‐atom site is expected to obtain bifunctional catalysts with ORR and OER activities. In addition, the morphology of the material will also affect the distribution (or exposure) of the active sites of the catalyst to a certain extent, so it is also one of the concerns. For example, zeolite imidazole frame (ZIF) material with large specific surface area is an excellent porous carrier.^[^ ^14^, ^27^ ^]^

Against the above background, here we try to find highly active bifunctional catalysts for ORR and OER through morphology regulation and construction of diatomic sites. Specifically, under the induction of surfactants, heteronuclear Fe and Co bimetal sites were successfully introduced into the starfish like ZIF framework (named FeCoNC/SL) to achieve efficient catalysis of ORR and the construction of alkaline Zn‐air cells with close to theoretical energy density. The half‐wave potential (*E*_1/2_) of the ORR with FeCoNC/SL participation reached 0.876 V, and the *E*_1/2_ of the commercial 20 wt% Pt/C, CoNC/SL, and FeNC/SL were only 0.866, 0.825, and 0.806 V versus RHE under the same conditions. At 0.4 V versus RHE, the kinetic current density (*J*_k_) of FeCoNC/SL reaches 4.21 mA cm^−2^, which is much higher than the 3.29 mA cm^−2^ of commercial 20 wt% Pt/C. More beneficially, the power density of the Zn‐air battery assembled with FeCoNC/SL material as the air cathode is as high as 224.8 mW cm^−2^, and the specific capacity is closer to the theoretical value, reaching 803 mAh g_Zn_^−1^. For ORR and Zn‐air batteries, FeCoNC/SL also exhibits fascinating electrochemical long‐lasting stability. These outstanding properties are all due to the high active site exposure ratio given by the bimetallic synergistic effect and the morphology engineering control carrier.

## 2 Results and Discussion

### 2.1 Morphology of Materials

**Figure 1:**
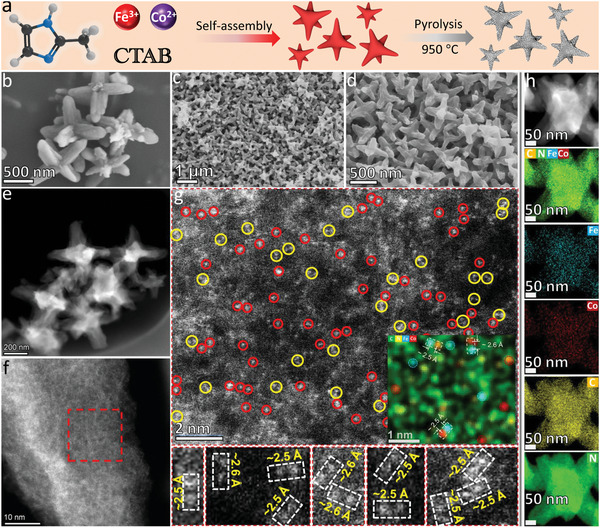
a) Schematic diagram of preparation of FeCoN5C/SL; b) SEM image of ZIF‐8/FeCo; c,d) SEM images of FeCoN5C/SL; e–g) TEM images (HAADF image model) of FeCoN5C/SL; and h) HAADF image and corresponding element (Fe, Co, C, and N element) mapping of FeCoN5C/SL.

**Figure** **1**a shows the preparation method of FeCoNC/SL with star‐like morphology, which involves the construction of porous metal‐organic framework, surface active agent control morphology, and in situ metal ion exchange technologies. Without the participation of the surfactant cetyltrimethylammonium bromide (CTAB), 2‐methylimidazole and Zn^2+^ will spontaneously self‐assemble into a regular dodecahedron‐shaped MOF (ZIF‐8) (Figure S1, Supporting Information). With the participation of the water‐soluble CTAB with long alkyl chain, the self‐assembly process of 2‐methylimidazole and Zn^2+^ will grow around the CTAB chain in an orderly manner, showing a star‐like morphology. Zn^2+^, Fe^3+^, and Co^2+^ have similar ion sizes and the coordination capacity of Fe^3+^ and Co^2+^ (common in six coordination) is higher than that of Zn^2+^ (common in four coordination), therefore, Fe^3+^ and Co^2+^ can easily replace the Zn^2+^ ion that coordinated with 2‐methylimidazole. When the pyrolysis temperature is higher than 906°C, the Zn in ZIF‐8 will evaporate and leave the ZIF‐8 matrix, leaving only the dodecahedron nitrogen–carbon (CN) framework, but Fe and Co will not leave the NC framework. Based on these principles, a CN material with a star‐like morphology doped with Fe and Co bimetal was successfully prepared. After pyrolysis treatment at 950 °C, the material still retains the star‐like morphology before pyrolysis, and the material shows a tendency of inward shrinkage only due to the departure of metal Zn and the increase of surface energy. In the scanning electron microscopy (SEM) and aberration corrected scanning transmission electron microscopy (AC‐TEM) images (Figure 1b–d), the FeCoNC/SL are all star‐like morphologies, with a size of about 500 nm. In the AC‐STEM images (Figure 1e,f) equipped with a high‐angle annular dark‐field (HAADF) mode, even if the scale was reduced to 10 nm (Figure 1f), it is still difficult to observe the nano‐sized bright spots in FeCoNC/SL, which represents no metal nanoparticles or metal nanoclusters are formed in FeCoNC/SL. When the scale is reduced to 2 nm (Figure 1g), a large number of atomic‐scale bright spots appear in the HAADF image. In the HAADF mode, the signals of elements with smaller atomic weights are weaker, and the signals of metal elements with larger atomic weights are stronger and easier to be collected, so the latter often appear as bright spots with high contrast.^[^ ^28^ ^]^ Although FeCoNC/SL does not contain metal nanoparticles (or nanoclusters), the signals of Fe and Co in FeCoNC/SL in element mapping are strong and evenly distributed (Figure 1h and Figure S2, Supporting Information). Based on this, it can be determined that the high‐contrast bright spots of the atomic size distribution in Figure 1g are the signals of metal atoms (Fe or Co). Note that some of these high‐contrast bright spots combine with each other to form atomic pairs with a pitch of about 0.25 or 0.26 nm. Element mapping imaging not only presents the distribution of different elements in the material, but also reflects the pairing of Fe and Co atoms (Figure S3, Supporting Information, and Figure 1g (inset)). By comparing the distance between atoms, it is considered that the diatomic pair in Figure 1g is Fe—Co diatomic.

It is difficult to find black nanoparticles or nanoclusters in TEM imaging of FeNC/SL and CoNC/SL, and no white nanoparticles or nanoclusters are found in AC‐STEM imaging (Figures S4a–f and S5a–f, Supporting Information). Element mapping shows that Fe exists in a highly dispersed state in FeNC/SL (Figure S4g–i, Supporting Information) and Co in CoNC/SL (Figure S5g–i, Supporting Information), and there is no aggregation state. Therefore, Fe and Co in FeNC/SL and CoNC/SL most likely exist in the form of single atoms.

The preparation method of FeCoNC/DL is the same as that of FeCoNC/SL except that CTAB was not added. SEM imaging (Figure S6, Supporting Information) shows that the morphology of FeCoNC/DL is similar to that of ZIF‐8, with a dodecahedral configuration. In AC‐STEM imaging (Figure S7, Supporting Information), it is difficult to detect high‐brightness nanoparticles or nanoclusters in HAADF mode. However, white bright spots of the atomic size distribution can be found in the field of view, and these bright spots come from the contributions of atoms with high atomic numbers. Elemental mapping imaging (Figure S7, Supporting Information) shows that the bright spots of atomic size distribution in HAADF mode come from the contribution of Fe and Co single atoms.

We found that the star‐like morphology of FeCoNC/SL originated from the regulation of CTAB. This is because in the presence of CTAB, both FeNC/SL (Figure S8, Supporting Information) and CoNC/SL (Figure S9, Supporting Information) exhibit star‐shaped morphologies, while the FeCoNC/DL obtained without CTAB has no star‐shaped features.

### 2.2 Physicochemical Status of Materials

**Figure 2:**
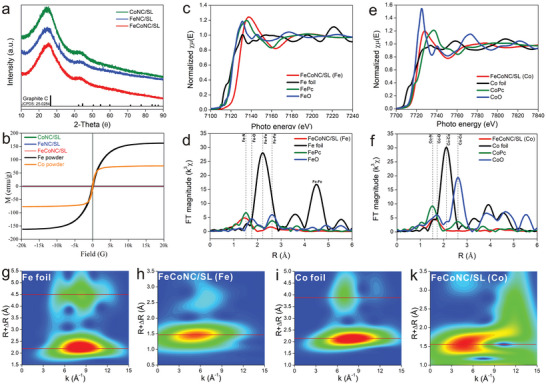
a) PXRD signals of CoNC/SL, FeNC/SL, and FeCoNC/SL; b) the hysteresis loop of FeCoNC/SL, FeNC/SL, CoNC/SL, Fe powder, and Co powder; c) XANES signals of FeCoNC/SL, Fe foil, FePc, and FeO; d) EXAFS signals of FeCoNC/SL, Fe foil, FePc, and FeO; e) XANES signals of FeCoN5C/SL, Co foil, CoPc, and CoO; f) EXAFS signals of FeCoNC/SL, Co foil, CoPc, and CoO; g–k) 2D color patch image obtained after wavelet transform processing R space, where (g) is Fe foil, (h) is FeCoNC/SL (detection element is Fe), (i) is Co foil, and (k) is FeCoNC/SL (detection element is Co).

To verify the visual information, the physicochemical properties of FeCoNC/SL were further revealed from the coordination structure level. In the powder X‐ray diffraction (PXRD) spectrum, FeCoNC/SL, FeNC/SL, and CoNC/SL show consistent signal characteristics (**Figure** **2**a). Except for the signal peak containing only carbon (JCPDS: 25‐0204), it is almost difficult to find the signal peak of Fe, Co, or FeCo alloy. The possible situations where there is no signal peak of metal material in PXRD are as follows: 1) the metal in the metal‐based material exists in an amorphous form; 2) the metal in the metal‐based material exists in the form of a single atom; and 3) the metal size in the metal‐based material is too small, but in this case a broad peak will appear in the PXRD. As there are no metal species in the form of nanoparticles or nanoclusters in TEM imaging and AC‐STEM imaging, Fe or Co in FeCoNC/SL, FeNC/SL, and CoNC/SL are all in the form of single atoms. This results in no peaks for crystalline metals in PXRD, nor the broad peaks that occur when metal species exist in amorphous form. This hypothesis is also verified by the hysteresis loop signal (Figure 2b), because Fe and Co exhibit magnetic properties only when they are present as lumps or nanoparticles (not monatomic forms), while they are not magnetic when they are present as monatomic forms. Furthermore, the synchrotron radiation accelerator‐based X‐ray absorption near‐edge structure (XANES) and extended X‐ray absorption fine structure (EXAFS) were used to reveal the valence state distribution and coordination structure of Fe and Co in FeCoNC/SL (Figure 2c–f). In XANES, the signal of FeCoNC/SL has no similarity with that of Fe foil and is slightly different from that of FePc and FeO. Therefore, the structure of FeCoNC/SL is different from that of Fe foil, FePc, and FeO (Figure 2c). At the edge of XANES, the photo energy of FeCoNC/SL is higher than Fe foil and is not between FePc (or FeO) and Fe foil, so the valence of Fe in FeCoNC/SL is greater than +2. Similarly, the signal of FeCoNC/SL in XANES is also different from Co foil, CoPc, and CoO, and the signal at edge also implies that the valence of Co in FeCoNC/SL is at least +2 (Figure 2e). As a verification, X‐ray photoelectron spectroscopy (XPS) also shows the valence state distribution of Fe and Co in FeCoNC/SL (Figure S10, Supporting Information). The XPS signal of Fe element in FeCoNC/SL is consistent with that of Fe element in FeNC/SL, and the binding energy of Fe2p_3/2_ signal peak is at 710.7 eV, so the valence of Fe is +3. Similarly, the XPS signal of Co element in FeCoNC/SL is consistent with that of Co element in CoNC/SL, and the binding energy of the Co2p_3/2_ signal peak is at 780.0 eV, so the valence of Co is +2. In EXAFS, Fe foil has strong signals at 2.2 and 4.5 Å, corresponding to the first and second shells of Fe—Fe bonds (Figure 2d).^[^ ^29^ ^]^ The difference is that the EXAFS of Fe in FeCoNC/SL has no Fe—Fe bond signal, but there is an obvious Fe—N bond signal (at 1.5 Å). Similarly, the EXAFS of Co foil showed strong signals of Co—Co bonds, where the signals at 2.15 and 3.9 Å correspond to the first and second shells of the Co—Co bond (Figure 2f). The EXAFS signal of Co in FeCoNC/SL also only contains the information of the Co—N bond (about 1.6 Å). It is well understood that Fe and Co in FeCoNC/SL coordinate with 2‐methylimidazole in the precursor ZIF‐8 (doped with Fe^3+^ and Co^2+^), so it is easy to form Fe—N and Co—N bonds in situ after pyrolysis. The 2D color patch image obtained by EXAFS after wavelet transformation has carried out a visual transformation of EXAFS (Figure 2g–k). Fe foil has high‐contrast color patches at 2.2 and 4.5 Å, which are the contribution of the Fe—Fe bond (Figure 2g). The high‐contrast color patches of Co foil at 2.15 and 3.9 Å are attributed to the contribution of the Co—Co bond (Figure 2i). In contrast, Fe and Co in FeCoNC/SL show only a single high‐contrast color patch (at 1.5 Å) in the wavelet transform graph (Figure 2h,k), consistent with the wavelet transform maps of FePc and CoPc (Figure S11, Supporting Information), respectively. Therefore, the bonding forms of Fe and Co in FeCoNC/SL are Fe—N and Co—N, respectively.

The HAADF image (Figure 1g) shows that FeCoNC/SL contains Fe and Co diatomic pairs, but EXAFS does not have the signal of Fe—Co bond, indicating that there is no chemical bond between Fe and Co atom pairs, and it is a heteronuclear bimetal. As it is difficult to accommodate both Fe and Co atoms in the N4 cavity of the phthalocyanine‐like structure, combining the distance between Fe and Co atoms, the most likely structure (FeCoN5C) was simulated by computer as shown in Figure S12a (Supporting Information, inset). The EXAFS (Figures S12a and S13, Supporting Information) and q space (Figure S12b, Supporting Information) signal fitted by the FeCoN5C structure is highly consistent with the experimentally collected signal, which verifies the existence of heteronuclear bimetals in the HAADF images. Except for the heteronuclear bimetal composed of Fe and Co, the structures of Fe and Co existing as single atoms are most likely to be FeN4C (corresponding to FeNC/SL) and CoN4C (corresponding to CoNC/SL). This is recognized by most research reports,^[^ ^30^ ^]^ and the EXAFS and q space signal simulated by the FeN4C and CoN4C structure also conforms to the results of the experimental collection to a certain extent (Figures S14 and S15, Supporting Information), Table S1 (Supporting Information) shows the fitted parameters. From the high‐resolution element mapping of FeCoNC/SL, the ratio of various metal sites can be roughly counted. Among them, the semiquantitative ratio of single‐atom Fe, single‐atom Co and FeCo heteronuclear diatoms is 9:15:5 (Figure S16, Supporting Information). Therefore, FeCoNC/SL is composed of monoatomic Fe, monoatomic Co, and heteronuclear FeCo diatoms.

### 2.3 Catalytic Activity for Oxygen Reduction Reaction

**Figure 3:**
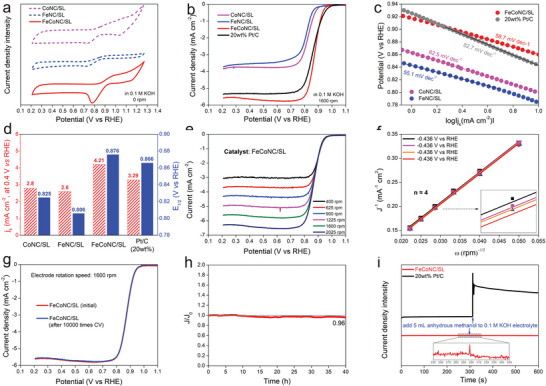
a) CV curves of FeN4C/SL, CoN4C/SL, and FeCoN5C/SL collected after the electrolyte was saturated with O_2_; b) LSV curves of FeN4C/SL, CoN4C/SL, FeCoN5C/SL, and 20 wt% Pt/C collected after the electrolyte was saturated with O_2_ (disk electrode rotation speed is 1600 rpm); c) Tafel curves converted from LSV signal; d) half‐wave potential of the ORR driven by CoN4C/SL, FeN4C/SL, FeCoN5C/SL, and 20 wt% Pt/C and the current density when the potential is 0.84 V; e) LSV signals collected by FeCoN5C/SL in an electrolyte saturated with O_2_ when the disk electrodes are at different rotation speeds; f) the relationship between *J* ^−1^ and speed *w* ^−1/2^ obtained according to the Koutecky–Levich equation; g) LSV curve of FeCoN5C/SL after 10 000 accelerated cyclic voltammetry experiments; h) durability of FeCoN5C/SL in long‐time running chrono‐ampere test; and i) anti‐methanol poisoning performance of FeCoN5C/SL and 20 wt% Pt/C.

As a proof‐of‐concept experiment, the performance of CoNC/SL, FeNC/SL, and FeCoNC/SL catalyzed ORR was evaluated in detail. In the electrolyte solution (0.1 m KOH) saturated with O_2_, an electrochemical reduction peak appeared in the cyclic voltammetry (CV) curves obtained by using CoNC/SL, FeNC/SL, and FeCoNC/SL as catalysts, respectively (**Figure** **3**a). When the electrolyte was saturated with N_2_, no reduction peak appeared in the CV curve (Figure S17, Supporting Information). Therefore, the reduction peak that appears is the signal peak of O_2_ being reduced. In other words, CoNC/SL, FeNC/SL, and FeCoNC/SL all have the ability to catalyze ORR. The difference is that the electrochemical reduction signal peak of O_2_ when FeCoNC/SL was used as a catalyst is much more obvious than when CoNC/SL and FeNC/SL were used as catalysts, respectively, and FeNC/SL has the worst response signal. The linear sweep voltammetry (LSV) curve shows a similar trend to that of CV. The half‐wave potential (*E*_1/2_) and limiting current density (*J*_L_) of the LSV curve obtained when FeCoNC/SL was used as the RDE electrode are both higher than the commercial platinum‐carbon catalyst (20 wt% Pt/C), reaching 0.876 V and ≈5.8 mA cm^−2^ (the RDE rotation speed is 1600 rpm) (Figure 3b). In contrast, the *E*_1/2_ and *J*_L_ of CoNC/SL and FeNC/SL are unsatisfactory. It should be noted that the LSV curves obtained when Ar and N_2_ saturate the electrolyte, respectively, almost overlap, so FeCoNC/SL has little ability to catalyze N_2_ reduction (Figure S18, Supporting Information). As the current density is extremely low without O_2_, the LSV curves involved in this paper have not been background subtracted. The slope of the Tafel curve obtained by converting the LSV curve near the starting potential reveals the rate of the ORR reaction kinetics. The Tafel slopes of 20 wt% Pt/C, FeNC/SL, CoNC/SL, and FeCoNC/SL are 82.7, 55.1, 62.5, and 58.7 mV dec^−1^, respectively, so FeCoNC/SL, CoNC/SL, and FeNC/SL all have faster kinetic reaction rates in driving ORR (Figure 3c). Kinetic current density (*J*_k_) as a comprehensive performance parameter is the best choice for evaluating ORR performance. When the potential is 0.4 V versus RHE, the *J*_k_ of FeCoNC/SL exceeds 20 wt% Pt/C catalyst (3.29 mA cm^−2^) and is much higher than CoNC/SL and FeNC/SL, reaching 4.21 mA cm^−2^ (Figure 3d). To clarify the reaction pathway of ORR, the LSV curve of ORR when the RDE electrode rotates at different speeds was collected. As the rotation speed of the RDE electrode increases, the *J*_L_ of the ORR driven by FeCoNC/SL continues to increase, but *E*_1/2_ remains almost unchanged (Figure 3e). After transforming Figure 3e into the data form of *J*_L_^−1^ varying with the rotation speed *w*(rpm)−1 through the Koutecky–Levich equation, the slopes of the straight lines obtained by the fitting are all close to 4 (Figure 3f). Therefore, driven by FeCoNC/SL, O_2_ is electrochemically reduced to H_2_O through a four‐electron process. In terms of electrochemical stability, FeCoNC/SL also showed excellent performance. After running 10 000 cycles of cyclic voltammetry, the LSV curve of the ORR driven by FeCoNC/SL almost coincides with the initial state (Figure 3g), which is difficult for most ORR catalysts to achieve. In addition, even if the chronocurrent experiment was continuously run for 40 h for a long time, the current density still reached 96% of the initial value (Figure 3h). As we are well‐known, Pt‐based materials are prone to lose ORR performance if they encounter alcohols in the process of catalyzing ORR. This is because Pt‐based materials are a kind of highly efficient electrochemical oxidation catalysts for alcohols. In this context, the performance of FeCoNC/SL as a methanol oxidation reaction (MOR) catalyst was evaluated. The results show that the current signal of the MOR driven by FeCoNC/SL is weak, so FeCoNC/SL is not an ideal MOR catalyst, but this result is beneficial for ORR. For example, after a sudden addition of methanol solution in running a chronoamperometric experiment, the current density driven by FeCoNC/SL is almost unaffected, but the ORR activity of Pt/C is severely hindered (Figure 3i). When used in ORR, PXRD (Figure S19, Supporting Information) and HAADF image (Figure S20, Supporting Information) show that there are still no metal nanoparticles or nanoclusters in FeCoNC/SL. These phenomena have verified that FeCoNC/SL is an excellent ORR catalyst with electrochemical stability.

### 2.4 Catalytic Activity for Oxygen Evolution Reaction

**Figure 4:**
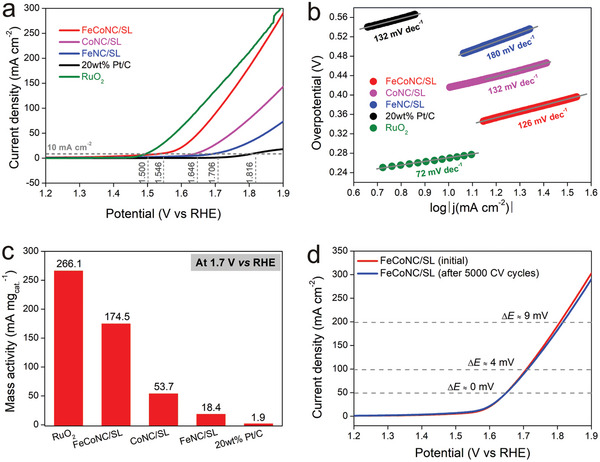
a) LSV curves collected when FeCoN5/SL was used as the working electrode; b) Tafel curves converted from LSV curves; c) the mass activity (applied potential is 1.7 V) of OER driven by FeCoN5C/SL, CoN4C/SL, FeN4C/SL, and 20 wt% Pt/C; and d) the stability of OER driven by FeCoN5C/SL.

As the reverse reaction of the ORR four‐electron reaction, the electrochemical oxygen evolution reaction (OER) is also important. This is because in practical applications, a reversible operation of a certain reaction is required to realize the reinitialization of the device, such as in the field of metal‐air batteries. Here, the activity characteristics of FeCoNC/SL as OER catalyst were evaluated in detail. In the LSV curve (**Figure** **4**a), the current density of OER driven by FeCoNC/SL is only slightly lower than that of commercial RuO_2_, the OER activity is much higher than that of CoNC/SL and FeNC/SL, and the overpotential is only 316 mV (at 10 mA cm^−2^). This phenomenon indicates that the co‐doping of Fe and Co has higher OER activity than single metal doping. In addition, FeCoNC/SL can obtain a lower Tafel slope when used as OER catalyst (Figure 4b), which represents the realization of the maximization of the kinetic reaction rate. Among the mass activity (at 1.7 V vs RHE) parameters obtained when normalizing the catalyst mass, the values ​​of RuO_2_, FeCoNC/SL, CoNC/SL, and FeNC/SL are 266.1, 174.5, 53.7, and 18.4 mA mg_cat._^−1^, respectively, Pt/C has the lowest activity, only 1.9 mA mg_cat._^−1^ (Figure 4c). Although the mass activity of RuO_2_ is higher than that of FeCoNC/SL, the mass ratio of Ru in RuO_2_ reaches 75.9 wt%, and the metal loading in FeCoNC/SL is only about 0.94 wt%. In comparison, the cost‐effectiveness ratio of FeCoNC/SL is lower. In addition, although the Pt/C catalyst exhibits excellent catalytic activity in ORR, it is not an ideal OER catalyst. The reason is that the high potential in the OER process can easily oxidize Pt into nonconductive PtOx species, which hinders the subsequent charge transfer process. In addition, PtOx can be reduced to zero‐valent Pt only when a relatively negative reduction potential (0.8 V vs RHE) is given. In other words, the oxidation thermodynamics of Pt within the OER window is irreversible. In contrast, Fe and Co in FeCoNC/SL existing in a high‐valence state can withstand the erosion of the high oxidation potential of OER. For example, after running 5000 cycles of cyclic voltammetry, the collected LSV curve is similar to the initial state, and the potential difference when the current density reaches 50, 100, and 200 mA cm^−2^ is only 0, 4, and 9 mV (Figure 4d), which verify that FeCoNC/SL also has durable electrochemical OER stability. Based on this, FeCoNC/SL has exhibited excellent bifunctional activity, which is highly competitive with other reported catalysts (Table S2, Supporting Information).

### 2.5 Performance of Zinc‐Air Batteries When the Material Is Used as an Air Cathode

**Figure 5:**
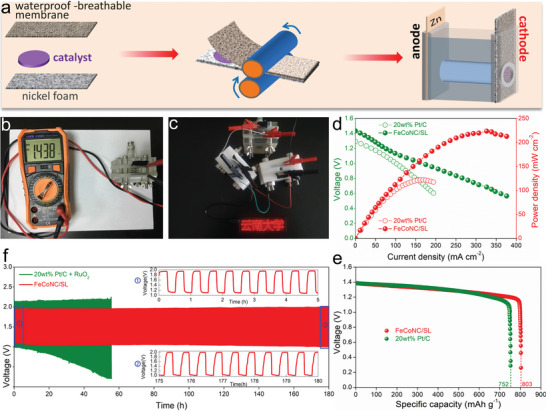
a) Schematic diagram of the assembly of a Zn‐air battery with a high‐purity Zn sheet as the anode and FeCoN5C/SL as the air cathode; b) the open‐circuit voltage of Zn‐air when FeCoN5C/SL was used as an air cathode; c) connecting three Zn‐air batteries in series (FeCoN5C/SL as the air cathode) can light up an LED array with a rated power of 5 V; d) LSV curve and corresponding power density of Zn‐air battery; e) specific capacity of Zn‐air battery; and f) long‐term charge and discharge test.

As verification, the performance of zinc‐air battery when FeCoNC/SL was used as an air cathode was explored to evaluate its practical application potential. Assemble the zinc‐air battery according to the preparation scheme in **Figure** **5**a, where the positive electrode is an air electrode containing FeCoNC/SL, the negative electrode is a high‐purity zinc sheet, and the electrolyte is a 6 m KOH solution containing 2 m Zn(AcO)_2_. When the FeCoNC/SL input is 10 mg and the geometric area of ​​the air cathode is about 1 cm^2^, the open‐circuit voltage of the zinc‐air battery reaches 1.438 V (Figure 5b). After connecting three zinc‐air batteries in series, it can drive the LED panel with a rated voltage of 5 V to operate normally (Figure 5c). As two important parameters for evaluating batteries, the power density and specific capacity of FeCoNC/SL exceed those of commercial Pt/C catalysts, reaching 224.8 mW cm^−2^ (Figure 5d) and 803 mAh g_Zn_^−1^ (Figure 5e), respectively. Note that the power density and specific capacity of FeCoNC/SL involved in zinc‐air batteries have been better than most ORR catalysts that have been reported (Table S3, Supporting Information). Due to the ORR activity and OER activity at the same time, the zinc‐air battery with FeCoNC/SL air cathode exhibits excellent charge and discharge performance. Even with continuous charging and discharging for 180 h (360 cycles), the charging and discharging voltages of the zinc‐air battery with FeCoNC/SL air cathode remain almost unchanged (Figure 5f). The difference is that, as a model catalyst for ORR, when Pt/C/RuO_2_ catalyst was used as an air cathode, the discharge voltage of a zinc‐air battery is lower and requires a higher charging voltage. What is important is that since Pt is easily oxidized to PtO_x_ under high oxidation potential, as the number of charge and discharge increases, the zinc‐air battery is gradually deactivated, which is manifested by the continuous decrease of the discharge voltage and the continuous increase of the required charging voltage.

### 2.6 Analysis of Electrocatalytic Activity

Compared with single‐metal‐doped FeNC/SL and CoNC/SL, the ORR activity and OER activity of FeCoNC/SL containing bimetal have been greatly improved, so the synergistic mechanism here needs to be further explored. In Nyquist plots (Figure S21, Supporting Information), FeCoNC/SL, FeNC/SL, and CoNC/SL have close electron transfer resistance (*R*_ct_) values, so the electron transfer rates of the three are similar. The difference is that the diffusion coefficient of FeCoNC/SL is higher than that of FeNC/SL and CoNC/SL, which can promote the adsorption of O_2_ on the catalyst surface. In addition, the CV curves (in 0.1 m KOH solution) (Figure S22a–c, Supporting Information) of FeCoNC/SL, FeNC/SL, and CoNC/SL at different scan speeds were collected and converted into a graph of current degree versus time (*J*–scan rate) (Figure S22d, Supporting Information). The *J*–scan rate data show that the electric double‐layer (*C*_dl_) capacitance values of FeCoNC/SL, FeNC/SL, and CoNC/SL are 7.65, 0.925, and 1.42 mF cm^−2^, respectively. The larger the *C*_dl_ value, the more electrochemically active area. As the bimetallic bimetal can adjust the electronic structure of each other and affect the electronic atmosphere of the nearby NC, the effective electrochemically active area in FeCoNC/SL is larger than that in FeNC/SL and CoNC/SL.

**Figure 6:**
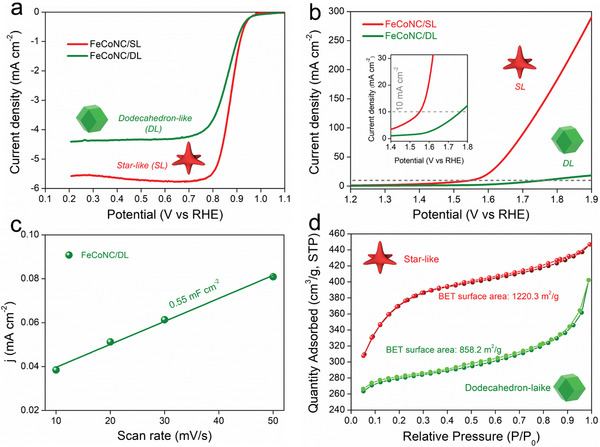
a) LSV curves of ORR (at 1600 rpm) when FeCoNC/SL and FeCoNC/DL were used as catalysts, respectively; b) LSV curves of ORR (at 1600 rpm) when FeCoNC/SL and FeCoNC/DL were used as catalysts, respectively; c) current density as a function of scanning rate (obtained by cyclic voltammetry); and d) BET surface areas of carbon–nitrogen materials with morphologies star‐like (red) and dodecahedron‐like (green), respectively.

As another important factor affecting the ORR activity, the effect of the morphological differences of the catalysts was evaluated in detail. In the case of the same dosage of Co and Fe, the half‐wave potential of the ORR driven by FeCoNC/SL as catalyst is at least about 16 mV higher than that when FeCoNC/DL was used as catalyst (**Figure** **6**a). The larger difference is manifested in the value of limiting current density (*j*_L_) of ORR, which is increased by about 33.8% for *j*_L_ when the morphology is transformed from dodecahedron‐like to star‐like. The difference in morphology also significantly affects the activity of OER. The OER overpotential is 316 mV (at 10 mA cm^−2^) when FeCoNC/SL was used as catalyst, while it reaches 533 mV when FeCoNC/DL was used as catalyst (Figure 6b). In general, the effect of the difference in morphology on the electrochemically active area is inevitable. In terms of *C*_dl_ value, the electrochemically active area of FeCoNC/SL with star‐like morphology is ≈14 times larger than that of dodecahedral FeCoNC/DL with nearly spherical morphology (Figure 6c). The significant effect of the difference in morphology is evident from the *C*_dl_ values, exposing the interior of the bulk material can increase the specific surface area of the catalyst and thus more reaction sites. Nitrogen adsorption/desorption tests confirmed the above inferences, and the CTAB‐involved morphology control engineering exposed more surface sites. The BET surface area of the star‐like carbon–nitrogen material is close to 1.5 times that of the dodecahedron‐like carbon–nitrogen material, reaching 1220.3 m^2^ g^−1^ (Figure 6d).

### 2.7 Density Functional Theory Analysis

**Figure 7:**
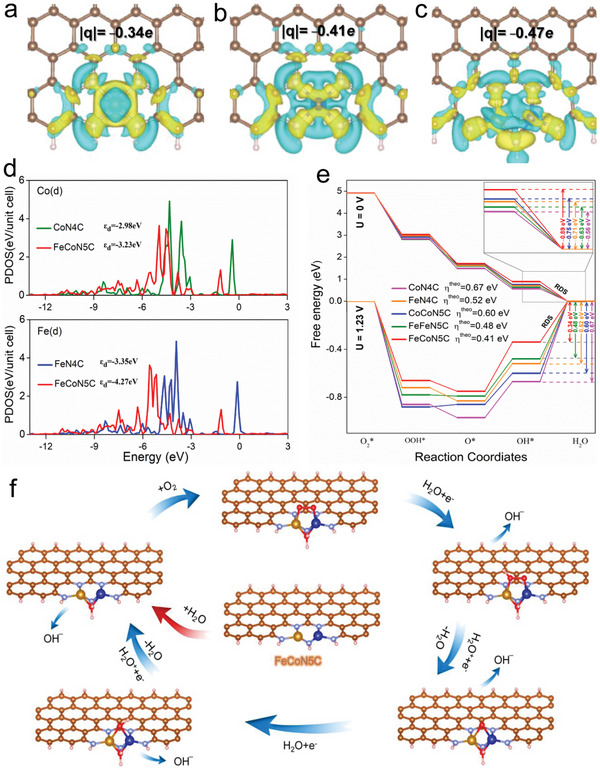
a–c) Structural models of FeN4C, CoN4C, and FeCoN5C after energy optimization; d) PDOS of Co elements in CoN4C and FeCoN5C, and PDOS of Fe elements in FeN4C and FeCoN5C; e) free energy step diagram of ORR (horizontal axis to the right) or OER (horizontal axis to the left) driven by FeCoN5C, FeFeN5C, CoCoN5C, FeN4C, and CoN4C, respectively; and f) the proposed ORR mechanism based on FeCoN5C catalysis.

Density functional theory (DFT) was further used to reveal the role of Fe—Co diatomic pairs in ORR and OER in FeCoNC/SL. Based on the HAADF images, XPS signal, EXAFS and their fitting results, a model (after optimization) for DFT calculation was constructed as shown in Figure S23a–c (Supporting Information). For comparison, the models FeFeN5C and CoCoN5C were also constructed (Figure S24, Supporting Information). It is worth noting that the structure of biatoms is easier to form (the formation energy is more negative) than the single metal atom configuration (Table S4, Supporting Information). The surface free energy of a single metal atom is large, so it is easy to spontaneously aggregate from a state where the atoms are dispersed. The formation of metal alloys is more favorable than the formation of M—N bonds, so there is a tendency for two metal atoms to approach each other, so the diatomic configuration is more favorable than the single‐atom configuration. In addition, the formation energy of the FeCoN5C model is more negative compared with the FeFeN5C and CoCoN5C configurations, indicating that the latter is more favorable for formation. We speculate that the formation of diatomic pairs may be more favorable than that of the same kind of atoms due to the charge transfer between different atoms. **Figure** **7**a shows the deformation charge density of FeN4C, CoN4C, and FeCoN5C, where the metal site showed a tendency to lose electrons. The electron loss of Co site and Fe site in CoN4C and FeN4C is about 0.41e and 0.34e, respectively, while the electron loss of FeCo heteronuclear diatomic site in FeCoN5C is up to 0.47e. Previous studies have shown that electron‐deficient (or positively charged) metal sites are beneficial to the adsorption of O_2_ and reaction intermediates.^[^ ^31^ ^]^ FeCoN5C loses more electrons, so it is more conducive to promote ORR smoothly, which is consistent with the experimental results. However, ORR and OER are a pair of mutually reversible reactions. To achieve high‐efficiency ORR and OER at the same time, the catalyst needs to have a moderate adsorption capacity for the intermediate.^[^ ^32^ ^]^ Generally, the d‐band center is an indicator for the binding strength between the metal center and reactants, the partial density of states (DOS) of Fe and Co is displayed in Figure 7d. Compared with the FeN4C single‐atom center site, the d‐band center of Fe shift to lower energy was found on the FeCoN5C diatomic center structure. Similar trend happened to the d‐band center of Co in FeCoN5C compared with CoN4C. The displacement of the d‐band center to the lower energy direction can weaken the adsorption capacity of FeCoN5C for intermediate species in ORR or OER, which is favorable for the escape of products from the catalyst surface.^[^ ^33^ ^]^ In FeCoN5C (Figure 7c), although the FeCo heteronuclear diatomic pair loss 0.47e, in terms of the local information of FeCo site, the Fe atom end showed a trend of electron loss, while the Co atom end showed a trend of electron injection, with Fe injecting about 0.12e into Co. Thus, in FeCoN5C, there is not only a transfer of electrons between the metal site and NC, but also a phenomenon of local charge redistribution between Fe and Co in the heteronuclear FeCo bimetallic pair. The electron shuttle effect enhances the reforming ability of electrons, which is beneficial to the smooth progress of electrocatalytic reactions.

It can be seen from the free energy step diagram that no matter the applied voltage is 0 or 1.23 V (Figure 7e), the potential determination step (PDS) from OH* to H_2_O in the ORR process on FeCoN5C is more likely to occur than FeN4C and CoN4C. Specifically, when the applied voltage is 0 V, the Gibbs free energy drop of the PDS in the ORR on FeCoN5C is more negative, which is a spontaneous process. When the voltage is 1.23 V, the PDS of ORR on FeCoN5C, CoN4C, and FeN4C all need to overcome a positive Gibbs free energy change, but FeCoN5C requires the smallest energy barrier. Unlike ORR, the PDS of OER in the reverse reaction is from OOH* to O_2_. Regardless of whether the applied voltage is 0 or 1.23 V, the PDS of the OER on FeCoN5C needs to overcome the smallest Gibbs free energy barrier, which is favorable for OER to occur. Through DFT analysis, the phenomenon produced in the experiment and the advantages of FeCoN5C structure are well revealed. O_2_ activation is a key step toward ORR.^[^ ^23^ ^]^ We note that at *U* = 0 V and *U* = 1.23 V, the formation of the heteronuclear FeCoN5C biatomic pair configuration is more favorable for PDS of ORR than the formation of FeFeN4C or CoCoN4C. Another noteworthy result is that the change of Fe species from FeN4C form to FeFeN5C or Co species from CoN4C form to CoCoN5C is overall favorable for ORR, suggesting that the formation of diatomic centers can promote ORR. As shown in the Figure S25 (Supporting Information), the bond length of O—O in optimized configurations of FeN4C, CoN4C, and FeCoN5C is 1.440, 1.467, and 1.485 Å, respectively, which clearly reveals that O_2_ activation is easier to be conducted on the surface of FeCoN5C catalyst. Meanwhile, adsorption energy (*E*_ads_) of *O_2_ on the Co sites of FeCoN5C, Fe sites of FeCoN5C, Co sites of CoN4C, and Fe sites of FeN4C are −3.58, −3.51, −3.35, and −3.22 eV, respectively, displaying high concentration O_2_ covered on the surface of FeCoN5C. In addition, the catalytic ORR efficiencies of FeCoN5C, CoN4C, and FeN4C showed a trend of FeCoN5C > FeN4C > CoN4C, and the theoretical overpotential (*η* ^theo^) were 0.41, 0.52, and 0.67 eV (Figure 7e), respectively, which indicated that the interdoping of Fe and Co could improve the overall activity of the catalyst.^[^ ^24^ ^]^

DFT results obviously elucidated the superiority of the FeCoN5C to catalyze ORR, on which the binding strength of ORR intermediates was weakened and therefore accelerated O—O breaking, which was responsible for the boosted ORR activity. Finally, the proposed mechanism of ORR based on FeCoN5C is depicted in Figure 7f.

It should be noted that most parameters in DFT calculations show that FeN4C is more beneficial to ORR and OER than CoN4C, but CoNC/SL showed better performance in experiments. We believe that this difference may come from the difference in the loading of active metals. The loading of Fe in FeNC/SL is about 0.79 wt%, while that of Co in CoNC/SL is about 1.01 wt%. Therefore, the loading (molar loading) of Co is about 23.4% higher than that of Fe. As DFT calculations only focus on single metal atoms, CoNC/SL contains more metal sites than FeNC/SL and thus exhibits better performance.

## 3 Conclusions

Using ZIF‐8 as the precursor, morphology control engineering and coordination atom exchange strategies were used to effectively embed heteronuclear Fe—Co bimetals on nitrogen‐doped carbon materials. The FeCoNC/SL obtained after annealing treatment shows excellent dual‐functional activity of oxygen reduction reaction and oxygen evolution reaction. In alkaline medium, the half‐wave potential and kinetic current density of the ORR driven by FeCoNC/SL surpassed the commercial Pt/C (20 wt%) catalyst, reaching 0.876 V and 4.21 mA cm^−2^, respectively. The overpotential of OER driven by FeCoNC/SL is as low as 316 mV (at 10 mA cm^−2^), and the mass activity is at least 3.2 and 9.4 times that of mononuclear CoNC/SL and FeNC/SL, respectively. When FeCoN5C/SL was used as the air cathode of Zn‐air battery, the power density, specific capacity and open circuit voltage are as high as 224.8 mW cm^−2^, 803 mAh g^−1^, and 1.438 V, respectively. Whether in ORR, OER, or Zn‐air batteries, FeCoN5C/SL has long‐lasting stability, which is beneficial for practical applications. DFT calculation shows that the formation of heteronuclear Fe—Co diatomic pairs can effectively regulate the electronic structure and improve the electron integration ability, which is also the main factor for FeCoNC/SL to have high ORR and OER bifunctional activities and achieve efficient operation of effective Zn‐Air batteries. In addition, converting the nearly spherical structure into a star‐shaped structure with a higher specific surface area allows more active sites to be exposed, which is the key to increasing the limiting current density.

## Conflict of Interest

The authors declare no conflict of interest.

## Supporting information

Supporting Information

## Data Availability

Research data are not shared.
