# Supplementary material for: Electron Modulation and Morphology Engineering Jointly Accelerate Oxygen Reaction to Enhance Zn‐Air Battery Performance
Source: Adv Sci (Weinh). 2023 Jan 22;10(8):2205889. doi: 10.1002/advs.202205889 (PMC10015884; doi:10.1002/advs.202205889)
Supplement: Supplementary file 1 — Supporting Information [file ADVS-10-2205889-s001.pdf]

ADVANCED SCIENCE
Open Access

Supporting Information

for *Adv. Sci.*, DOI 10.1002/advs.202205889

# Electron Modulation and Morphology Engineering Jointly Accelerate Oxygen Reaction to Enhance Zn-Air Battery Performance

*Xue Zhao, Jianbing Chen, Zenghui Bi, Songqing Chen, Ligang Feng*, Xiaohai Zhou, Haibo Zhang*, Yingtang Zhou*, Thomas Wågberg and Guangzhi Hu**

Supporting Information

**Electron modulation and morphology engineering jointly accelerate oxygen reaction to enhance Zn-air battery performance**

*Xue Zhao, Jianbing Chen, Zenghui Bi, Songqing Chen, Ligang Feng*, Xiaohai Zhou, Haibo Zhang*, Yingtang Zhou*, Thomas Wågberg, and Guangzhi Hu**

Supporting Information /Text

Experimental Method

*Materials and Methods*

Commercial 20 wt% Pt/C catalysts was purchased from Johnson Matthey, 5% Nafion solution was purchased from DuPont, and other chemicals were purchased from Aladdin Chemical Reagent Co., Ltd. and used as is. Carbon paper (hydrophilic) was purchased from Tianjin evs chemical Co. Ltd.., and foamed nickel (thickness: 1 mm) was purchased from Changde Liyuan New Material Co., Ltd.. The zinc-air battery module was purchased from Tianjin Aida Hengsheng Technology Co., Ltd.., and the area of the window where the catalyst was placed was 1 $cm^2$. The water quality used in the experiment was ultrapure water (18 $M\Omega\cdot cm^{-1}$).

*Catalyst preparation method*

First, 18 g of 2-methylimidazole and 40 mg of cetyltrimethylammonium bromide (CTAB) were dissolved in 320 mL of water as solution A. Then, 2.376 g $Zn(NO_3)_2\cdot6H_2O$, 40 mg $Co(NO_3)_2\cdot6H_2O$ and 52 mg $Fe(NO_3)_3\cdot9H_2O$ were dissolved in 80 mL of ultrapure water to form solution B. Subsequently, the B solution was quickly injected into the A solution under magnetic stirring (1200 rpm) and the stirring continued for 3 h. After the reaction, the filter cake was collected by filtration, washed alternately with ultrapure water and methanol 3 times, and dried in vacuum at 60 °C for 6 h. The obtained precursor was recorded as ZIF-8/FeCo. Finally, the FeCoNC/SL was obtained according to the following pyrolysis procedure: the initial temperature is 25 °C, the filling gas is Ar gas, the heating rate is 5 °C/min, the final temperature is 950 °C and kept for 3 h, and finally the temperature is naturally cooled.

The preparation method of ZIF-8 is as follows. First, 18 g of 2-methylimidazole was dissolved in 320 mL of water as solution A, and 2.376 g of Zn(NO3)26H2O was dissolved in 80 mL of ultrapure water to form solution B. Subsequently, solution B was rapidly injected into solution A under magnetic stirring (1200 rpm), and stirring was continued for 3 h. After

the reaction, the filter cake was collected by filtration, washed alternately with ultrapure water and methanol three times, and then vacuum-dried at 60 °C for 6 h to obtain ZIF-8. Nitrogen-doped carbon material (named NC) was obtained by treating ZIF-8 according to the heat treatment procedure for preparing FeCoNC/SL.

The preparation method of FeCoNC/DL is the same as that of FeCoNC/SL except that CTAB was not added.

The method of preparing FeNC/SL and CoNC/SL was the same as the method of preparing FeCoNC/SL, only the following modifications were made:

When preparing FeNC/SL, $Co(NO_3)_2 \cdot 6H_2O$ was not introduced and the input amount of $Fe(NO_3)_2 \cdot 9H_2O$ is 104 mg. $Fe(NO_3)_2 \cdot 9H_2O$ was not introduced when preparing CoNC/SL, and the input amount of $Co(NO_3)_2 \cdot 6H_2O$ is 80 mg.

*Characterization method*

The morphology of the material was collected by a field emission scanning electron microscopy (SEM, Zeiss Sigma300), a transmission electron microscopy (TF20), and an aberration corrected scanning transmission electron microscope (AC-STEM, FEI Themis Z) equipped with a spherical aberration corrector and an energy dispersion component. The powder X-ray diffraction signal was collected on an X-ray diffractometer named Rigaku Ultima IV, where the anode target was a Cu target and the collection speed was 10°/min. X-ray absorption spectroscopy (XAS) was collected at the Taiwan Synchrotron Radiation Research Center (NSRRC), and the beam was TLS07A1. The XAS raw data was subjected to background subtraction, normalization, k3-weighted Fourier transform and wavelet transform by Athena software. X-ray photoelectron spectroscopy (XPS) signals were collected on an energy spectrometer named Thermo Scientific K-Alpha+, and the binding energy was corrected by standard carbon. The magnetic intensity of the material was collected on a hysteresis loop measuring instrument (LakeShore, 7404). The nitrogen adsorption and desorption experiment was collected on a fully automatic specific surface and porosity

analyzer named ASAP2020, where the pre-degassing temperature was 300 °C. The metal loading in the material was determined by inductively coupled plasma optical emission spectrometer (ICP-OES, Agilent 7700 s). Among them, 0.1 g of the sample was dispersed into 10 mL of aqua regia, and ultrasonically treated for 10 min to completely dissolve the metals in the material into aqua regia. Then, the residue was removed by filtration and the mixed solution was diluted 100 times for testing ICP-OES. In the results, the loadings of Co in CoNC/SL, Fe in FeNC/SL, Co in FeCoNC/SL and Fe in FeCoNC/SL are 1.01wt%, 0.79wt%, 0.53wt% and 0.41wt%, respectively (**Table S5**).

*Electrochemical experiment*

The electrochemical experiment was carried out in a single-chamber electrolytic cell equipped with a rotating disk electrode (RDE), and electrochemical signals were recorded via an electrochemical workstation (Metrohm Autolab). In the three-electrode system, the reference electrode is a Hg/HgO electrode filled with 1 M KOH solution, and the counter electrode is a high-purity graphite rod (purity 99.999wt%). The preparation method of the RDE as the working electrode is as follows. 10 mg of catalyst was fully ultrasonically dispersed in a 1 mL dispersion solution containing 650 μL isopropanol, 300 μL ultrapure water and 50 μL 5% Nafion to form a uniform ink. Then pipette 10 uL ink drop-coated on the 5 mm diameter glassy carbon electrode and air dry. Before starting the electrochemical experiment, first bubbling the electrolyte with $O_2$ for 1 h to saturate the dissolved oxygen in the electrolyte, and then run a 10-cycle cyclic voltammetry experiment (sweep rate of 100 mV/s) to stabilize the working electrode. The linear sweep voltammetry (LSV) curves of different catalysts were collected at a speed of 1600 rpm. Only when the electron transfer number was studied, the speed of the RDE was controlled to be 400, 625, 900, 1225, 1600 and 2025 rpm, respectively. Note: In consideration of the actual application environment and fair comparison between different reports, the potentials involved in this report are not *IR* compensated.

The zinc-air battery consists of a positive electrode and a negative electrode. The negative electrode is a high-purity zinc sheet (with a thickness of 0.5 mm), the positive electrode is an air cathode composed of foamed nickel, a catalyst and a water-proof and breathable membrane, and the battery filling liquid is a 6 M KOH solution containing 2 M $Zn(CH_3COO)_2$. The preparation process of the air cathode is as follows: 1) Thoroughly grind and mix 10 mg catalyst, 30 mg carbon black and 20 μL 5% Nafion solution; 2) Place the catalyst in the interlayer between the foamed nickel and the water-proof and breathable membrane (the effective area is about 1 $cm^2$), and then compact by a pair of rollers; 3) Place the foamed nickel side close to the battery filling fluid, and the water-proof and breathable membrane side to contact the air, assemble the air cathode into the Zn-air battery. When testing the specific capacity of zinc-air potential, the discharge current was set at 7.8 mA and the cut-off voltage was 0.1 V. The charge and discharge current density was set 10 mA, the discharge cut-off voltage was set to 0.5 V, and the charge cut-off voltage was set to 2.0 V.

The electron transfer number of ORR is calculated by the Koutecky-Levich equation, where the Koutecky-Levich equation is as follows:

$$\frac{1}{\mathrm{j}} = \frac{1}{j_k} + \frac{1}{j_L} = \frac{1}{j_L} + \frac{1}{B\omega^{1/2}} \qquad \text{Eq.S1}$$

$$B = 0.2nFD_0^{2/3}C_0v^{-1/6} \qquad \text{Eq.S2}$$

Where j, $j_k$, $j_L$ are current density, dynamic current density and limiting current density respectively; *w*, F, $C_0$, $D_0$ and *v* are the angular velocity of the working electrode, the Faraday constant (96485 C/mol), the number of electron transfer, the concentration of $O_2$ in the electrolyte, the diffusion coefficient of $O_2$ and the dynamic viscosity, respectively.

The electrode potential calibration procedure of the reference electrode was as follows. The reference electrode is Hg/HgO filled with 1M KOH solution, the working electrode and the counter electrode are Pt discs (diameter 3 mm), and the electrolyte is 1 M KOH solution saturated with $H_2$. The collected CV curves were shown in **Figure S26**, the potential at which

hydrogen evolution begins is about 0.96 V, which is 0 V vs RHE when converted to a reversible hydrogen electrode (vs RHE). Therefore, the electrode potential of the reference electrode in this study was 0.134 V.

The acquisition conditions of Nyquist plots were as follows: the electrolyte is a mixed solution containing 0.1 M $K_3Fe(CN)_6$ and 0.1 M $K_4Fe(CN)_6$; the reference electrode is a calomel electrode (filling solution is saturated potassium chloride); the counter electrode is high-purity graphite rod; the working electrode is a glassy carbon electrode (3 mm in diameter) coated with 10 μL ink (catalyst dispersion); the potential is set to open circuit voltage (automatically filled in by the instrument); the frequency range is set to $10^7$-0.1 Hz

*Density functional theory calculation methods*

Vienna *ab initio* Simulation PackageVASP with GGA-PBE functional was employed in DFT. DFT+U was employ with values of U-J parameters for Fe (5) and Co (3.42). The cutoff energy was set to be 500 eV with k point 2×2×1 for geometry optimizations. The convergence threshold was set to $10^{-5}$ eV in energy and 0.02 eV/Å in force, respectively. To prevent the interaction between two slabs, the vacuum layer thickness was set to 15 Å.

Three models were established including FeN4C, CoN4C and FeCoN5C. The lattice parameters for all of them are a=17.21650, b= 14.00000, c=17.00000 Å and with alpha=90°, beta=90°, gamma=90°.

The free energy of each elementary step in the reactions was demonstrated using the computational hydrogen electrode (CHE) model for oxygen reduction reaction (ORR). Considering the $O_2$ molecular is not broken before reduction, the associative 4e reduction pathway was evaluated to be most feasible for ORR in this work, as follows (Eq.S3-7):

$O_2 + * \rightarrow O_2*$ Eq.S3

$O_2* + H_2O + e^- \rightarrow OOH* + OH^-$ Eq.S4

$OOH* + e^- \rightarrow O* + OH^-$ Eq.S5

$O* + H_2O + e- \rightarrow OH* + OH^-$ Eq.S6

$OH^* + e^- \rightarrow OH^- + *$ Eq.S7

Where, * represents the active site on the corresponding surface

The free energy diagram was constructed according to:

$\Delta G = \Delta E + \Delta ZPE - T\Delta S - neU$ Eq.S8

Note: n refers to the electron number of the states. ΔE represents the reaction energy of each reaction step. ΔZPE and ΔS were obtained from the vibrational frequency calculations, which were computed through 0.015Å displacement of absorbents in three directions with the fixed slab.

The theoretical reaction overpotential ($\eta^{ORR}$) was evaluated to the difference between the minimum voltage needed for the ORR (1.23 V) and the voltage required for changing all the free-energy steps into downhill, which record as:

$\eta^{ORR} = 1.23 - \min(\Delta G1\text{-}4)$ Eq.S9

Where, min (ΔG1-4) is the step with the smallest ΔG value in ΔG1-4.

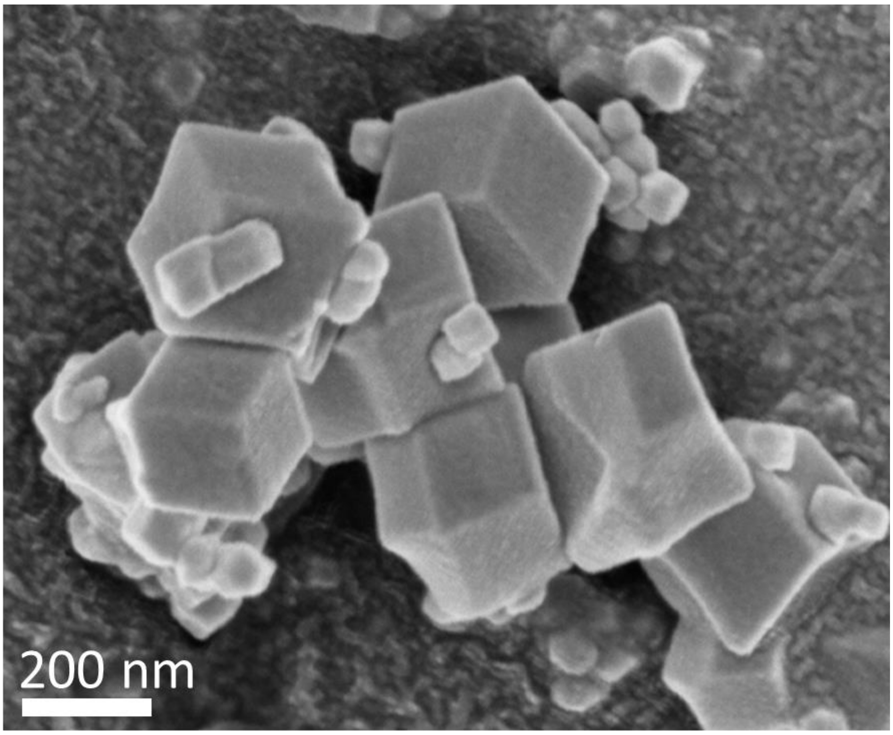


**Figure S1**. SEM image of ZIF-8

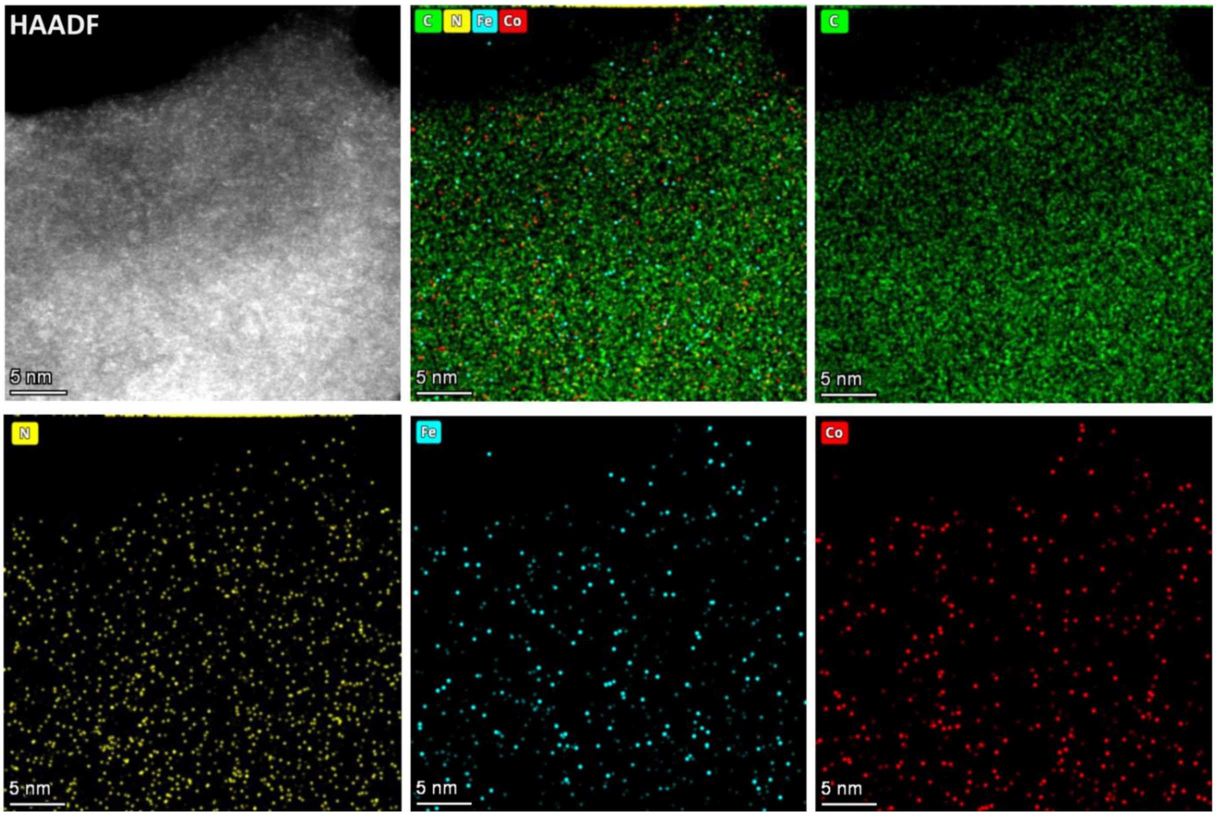


**Figure S2.** High-resolution elemental mapping images of FeCoNC/SL

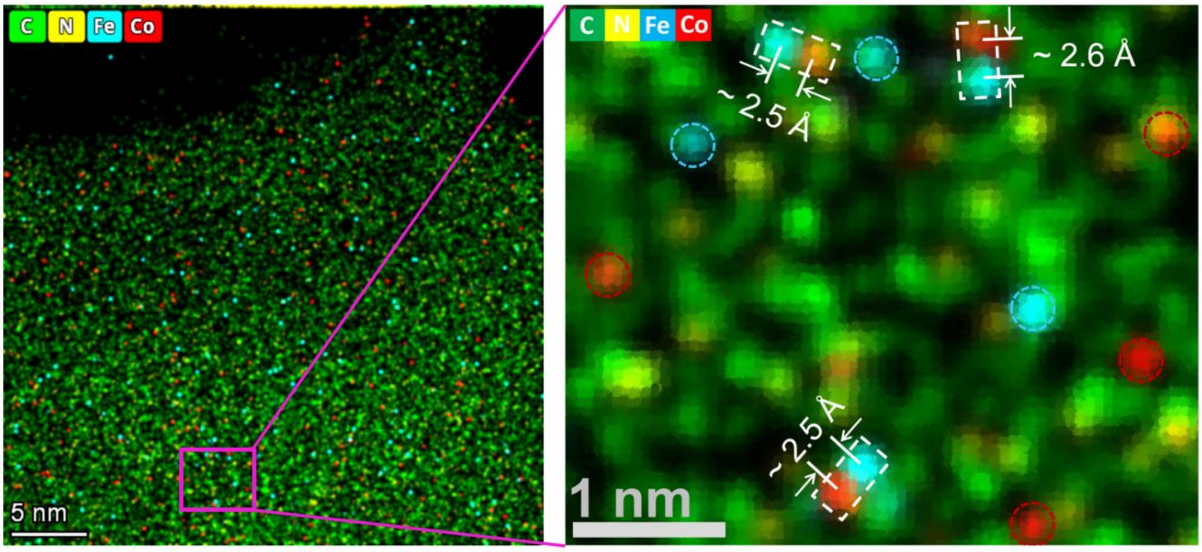


**Figure S3.** Fe-Co diatomic pair presented in the high-resolution elemental mapping images.

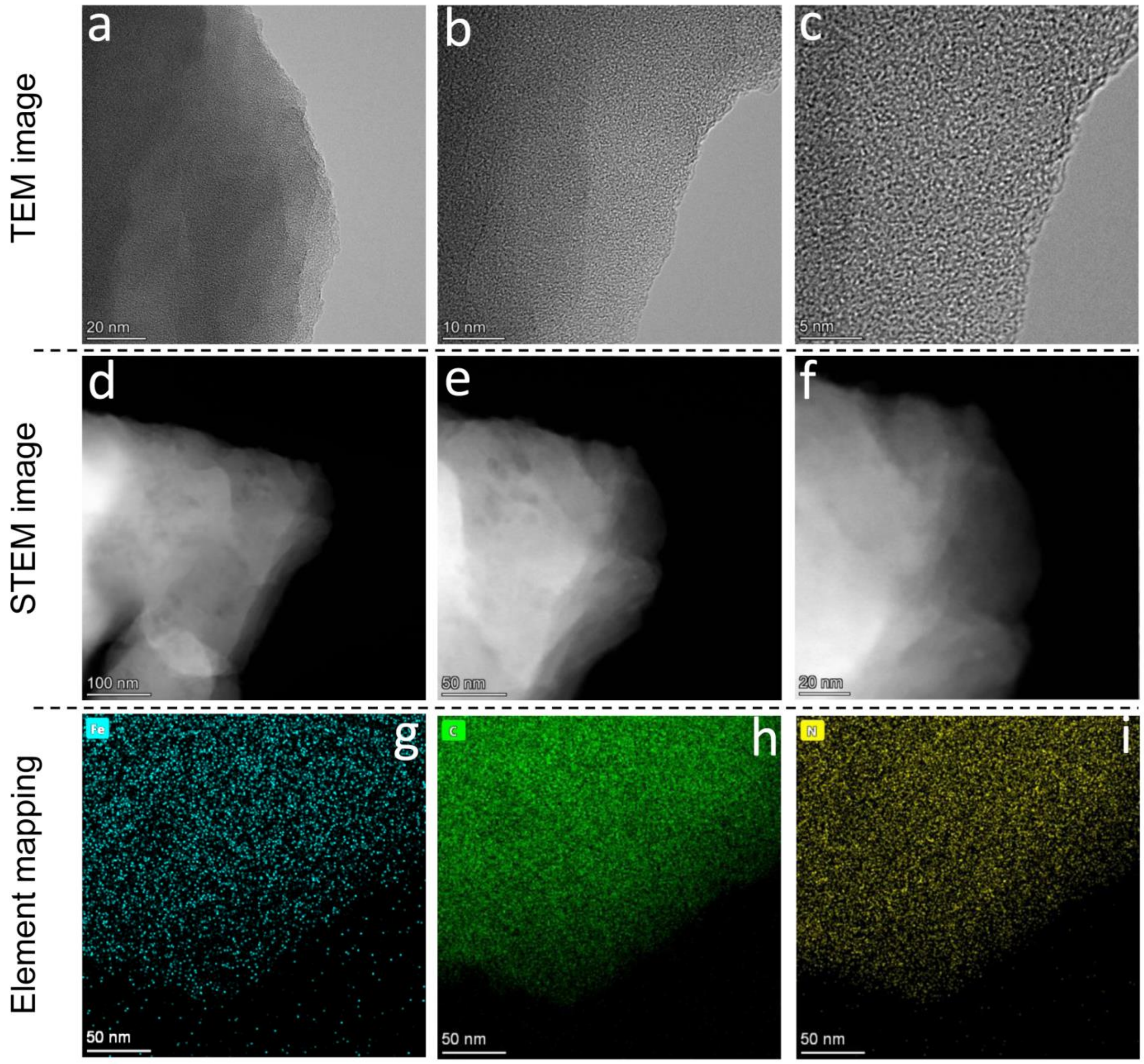


**Figure S4.** (a-c) TEM images of FeNC/SL; (d-f) AC-STEM images (HAADF mode) of FeNC/SL; (g-i) Element mapping of FeNC/SL

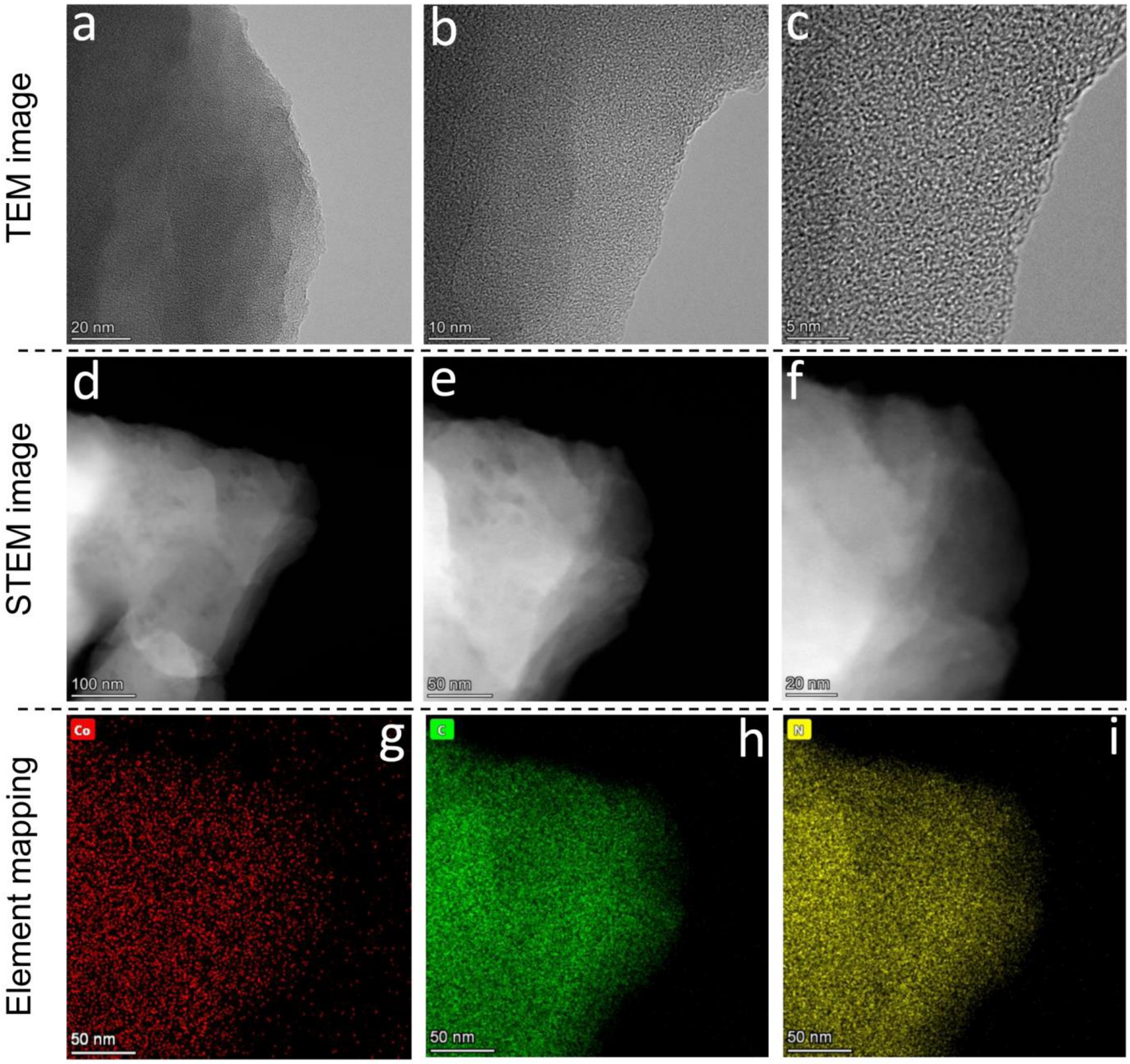


**Figure S5.** (a-c) TEM images of CoNC/SL; (d-f) AC-STEM images (HAADF mode) of CoNC/SL; (g-i) Element mapping of CoNC/SL

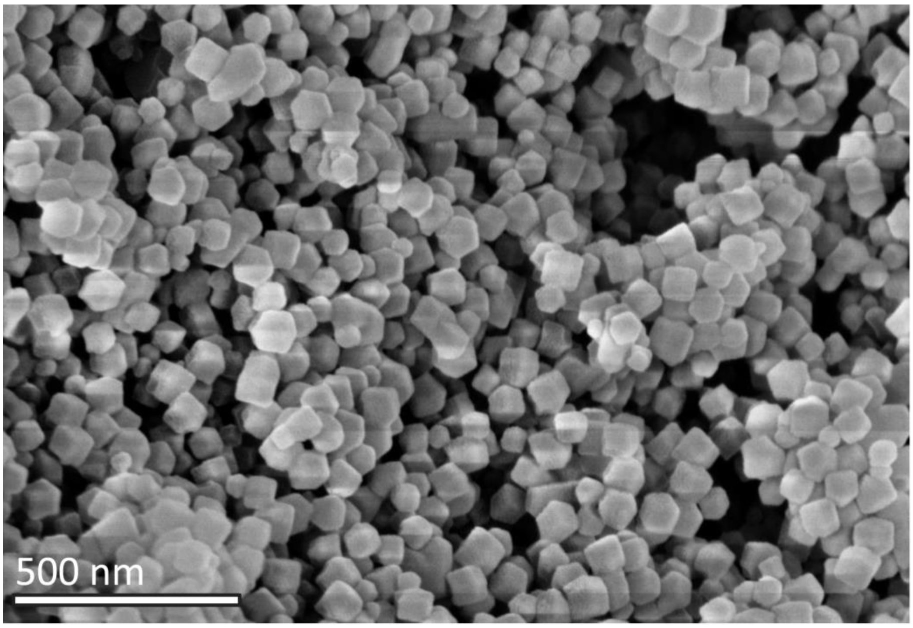


**Figure S6**. SEM image of FeCoNC/DL

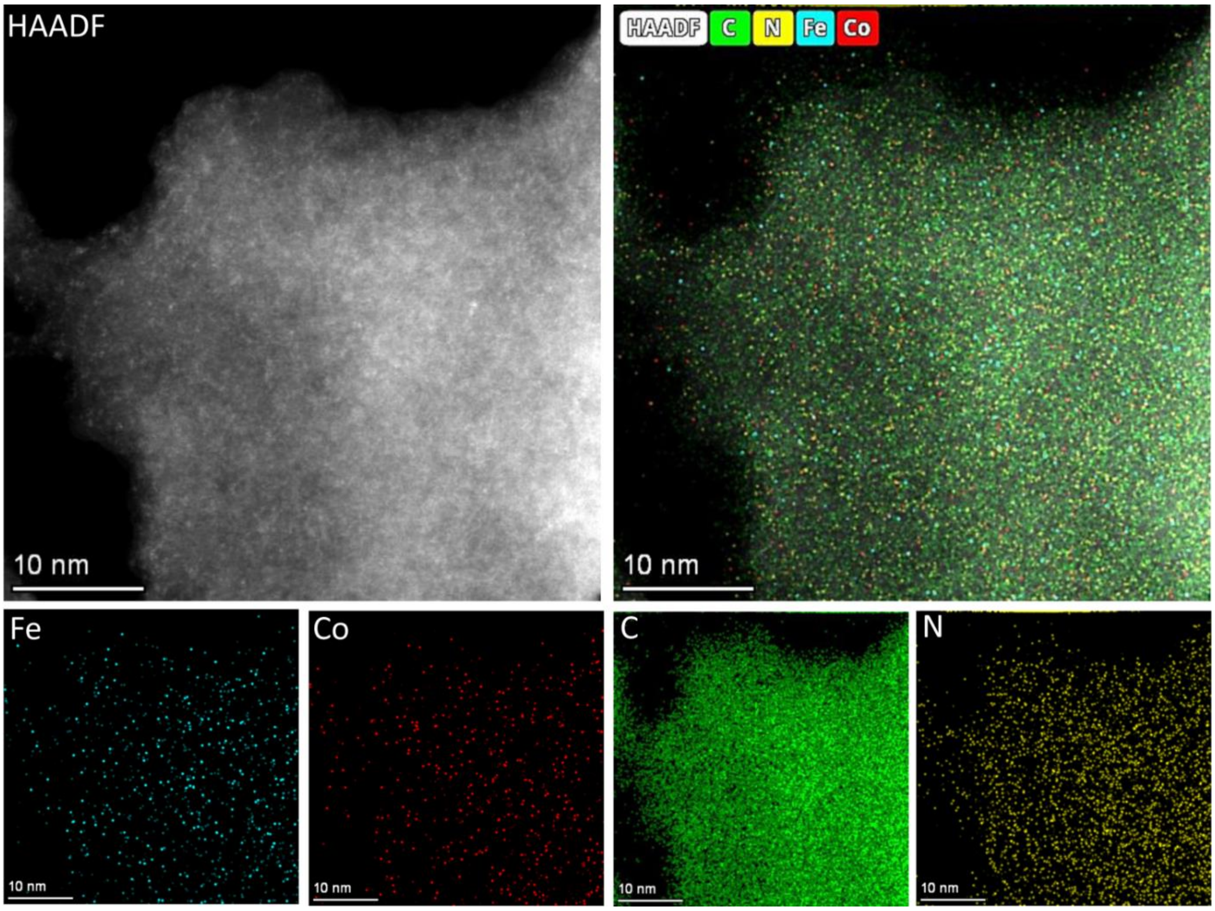


**Figure S7**. TEM image (HAADF mode) of FeCoNC/DL and corresponding elemental distribution imaging

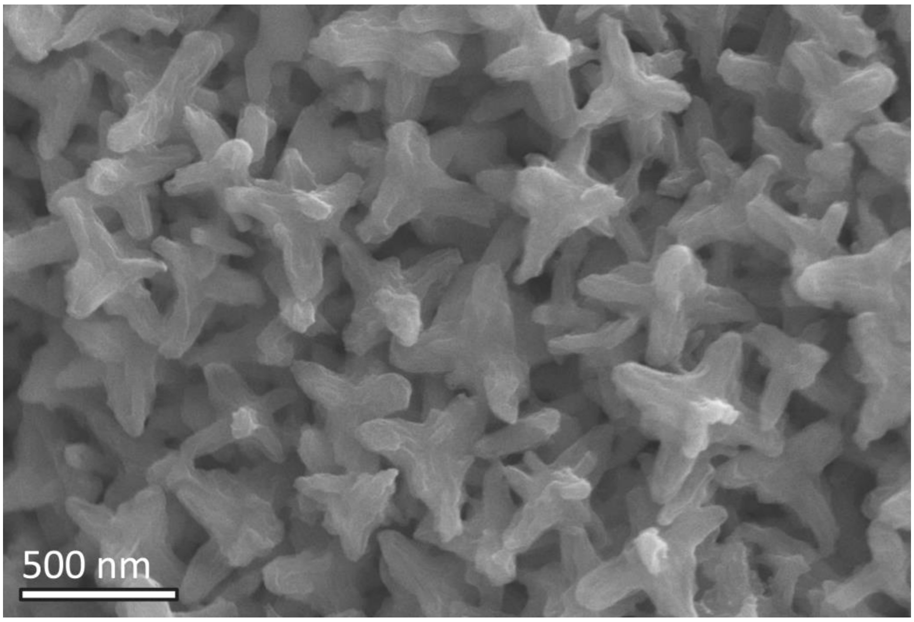


**Figure S8**. SEM image of FeNC/SL

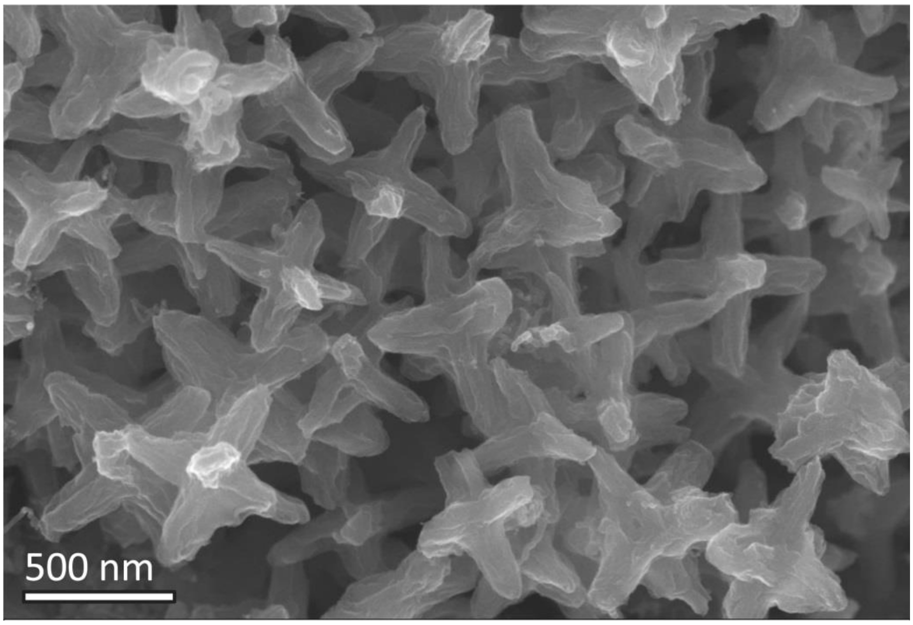

**Figure S9**. SEM image of CoNC/SL

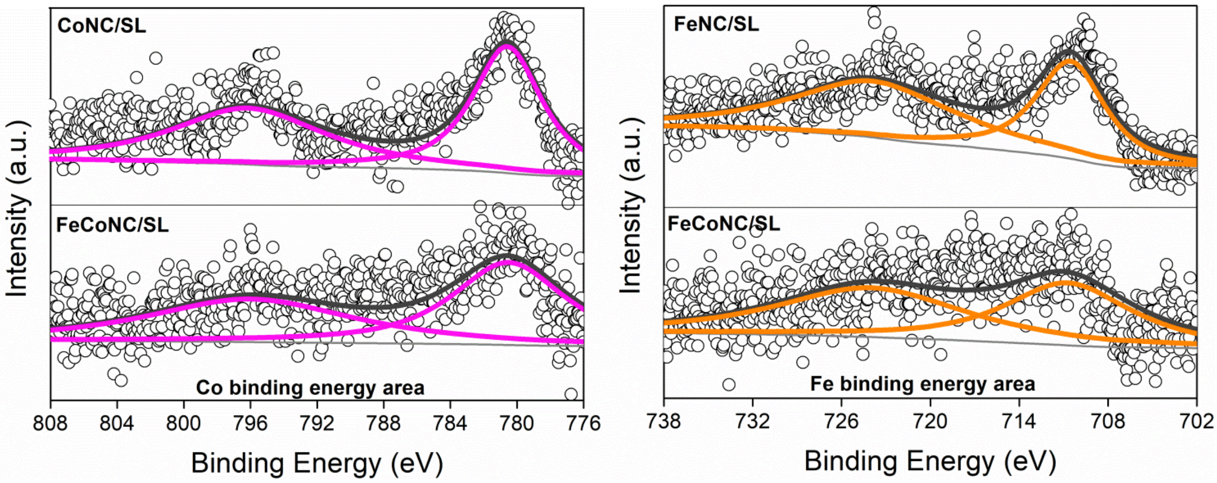


**Figure S10**. XPS of FeNC/SL, CoNC/SL, and FeCoNC/SL

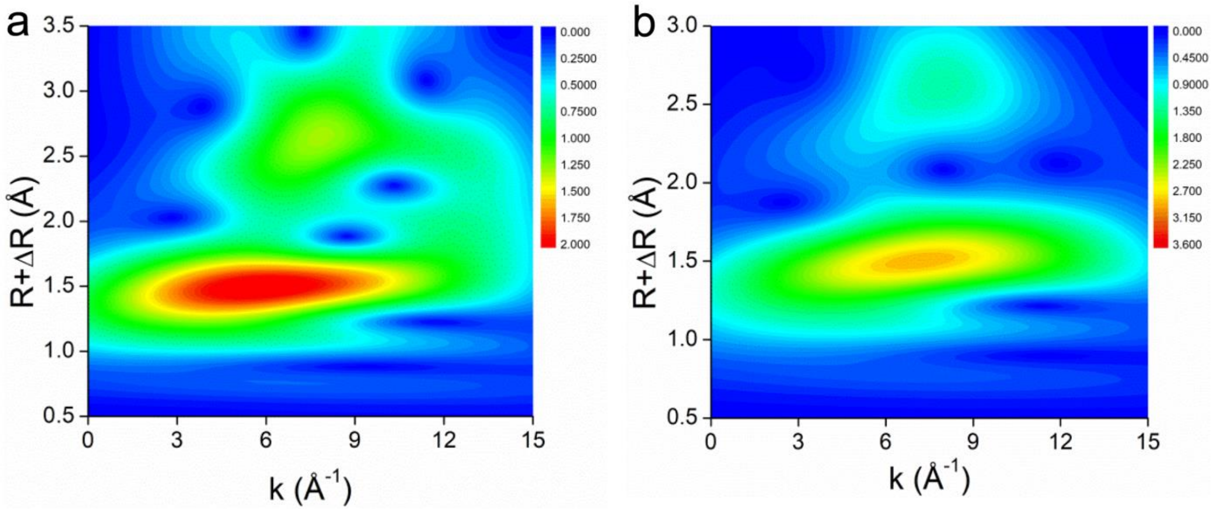


**Figure S11**. Two-dimensional color patch image obtained after wavelet transform processing R space, where a is FePc foil, b is CoPc.

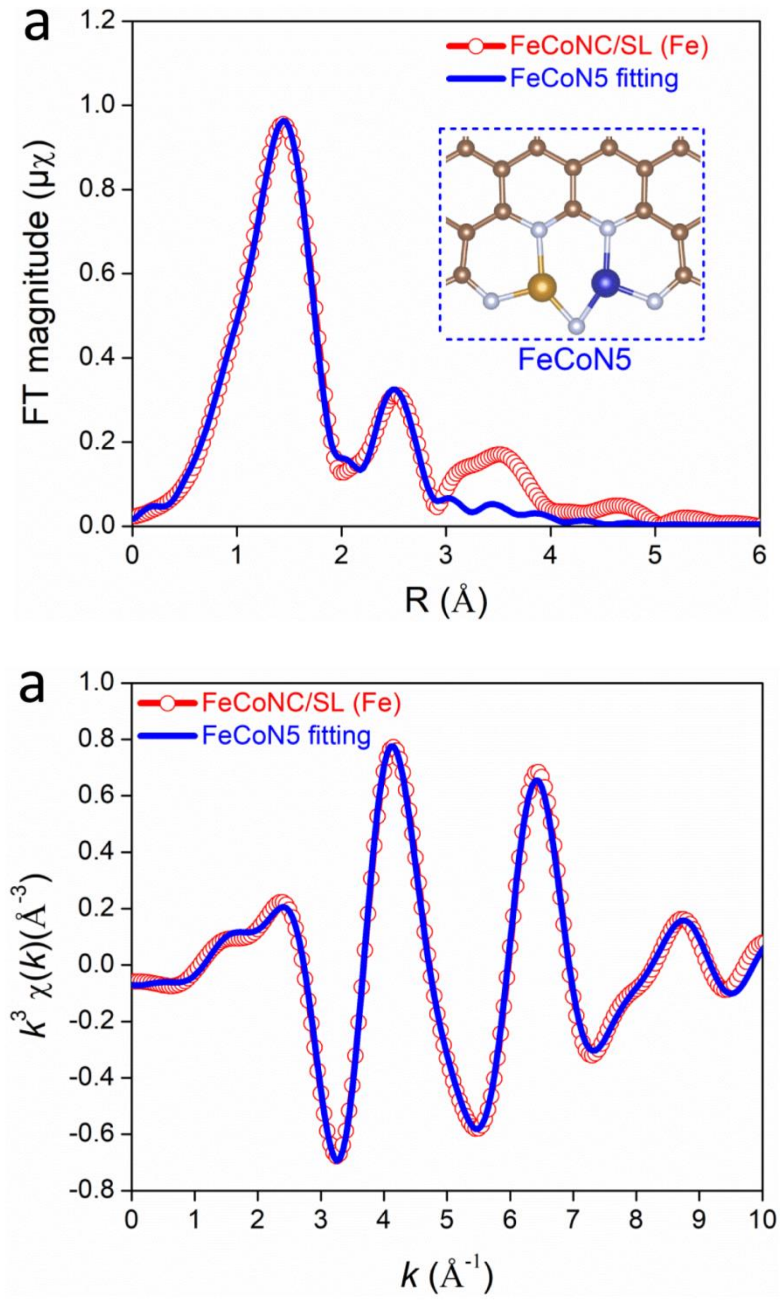


**Figure S12**. (a) EXAFS signal obtained by experimental collection (FeCoNC/SL) and theoretical simulation (FeCoN5C), respectively; (b) q space signal obtained by experimental collection (FeCoNC/SL) and theoretical simulation (FeCoN5C), respectively

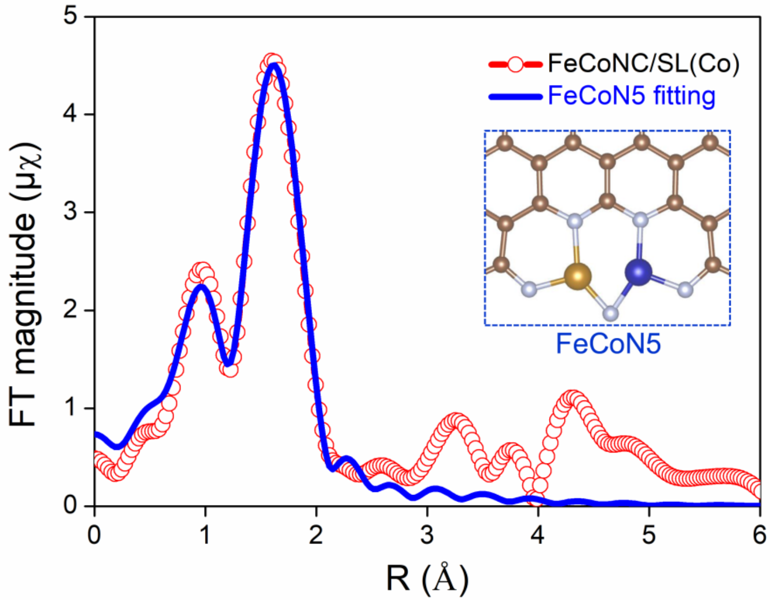


**Figure S13.** EXAFS signal obtained by experimental collection (FeCoNC/SL) and theoretical simulation (FeCoN5C), respectively

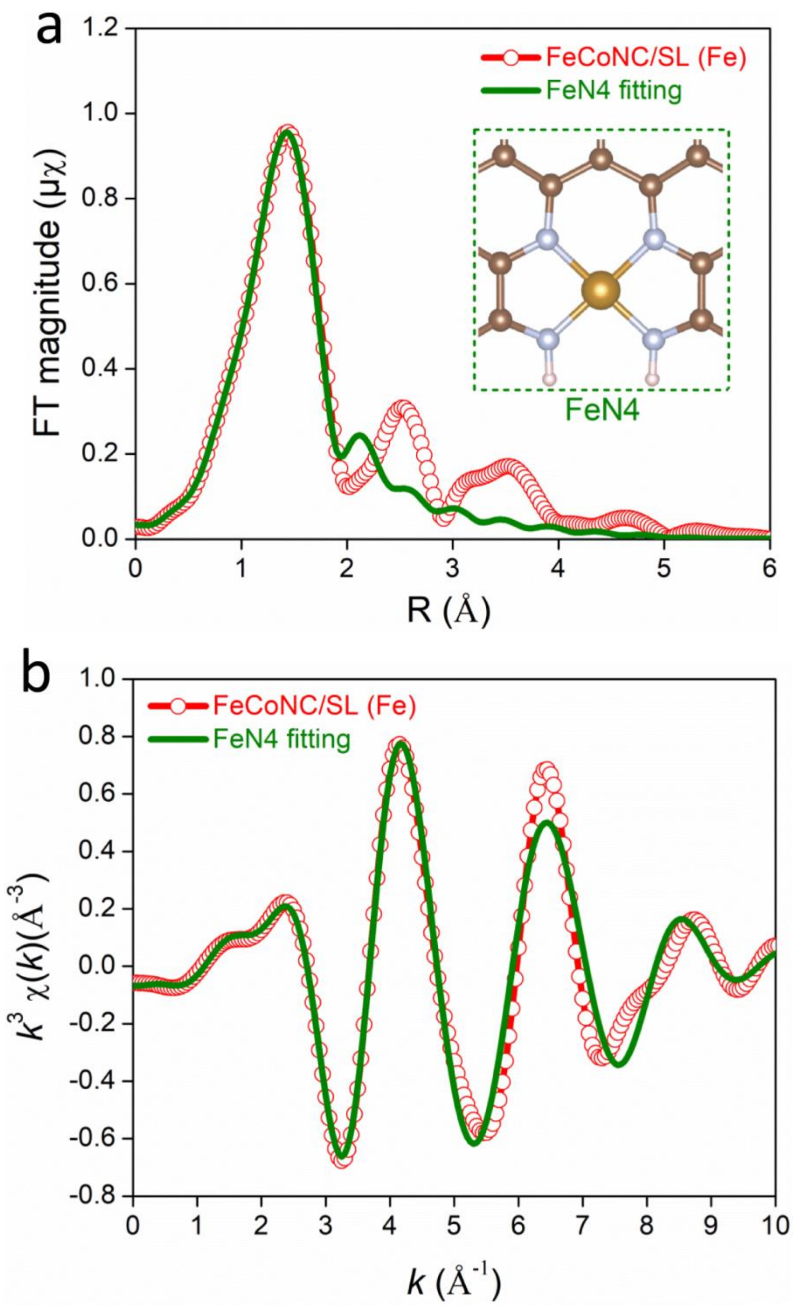


**Figure S14**. (a) EXAFS signal obtained by experimental collection (FeCoNC/SL) and theoretical simulation (FeN4C), respectively; (b) q space signal obtained by experimental collection (FeCoNC/SL) and theoretical simulation (FeN4C), respectively

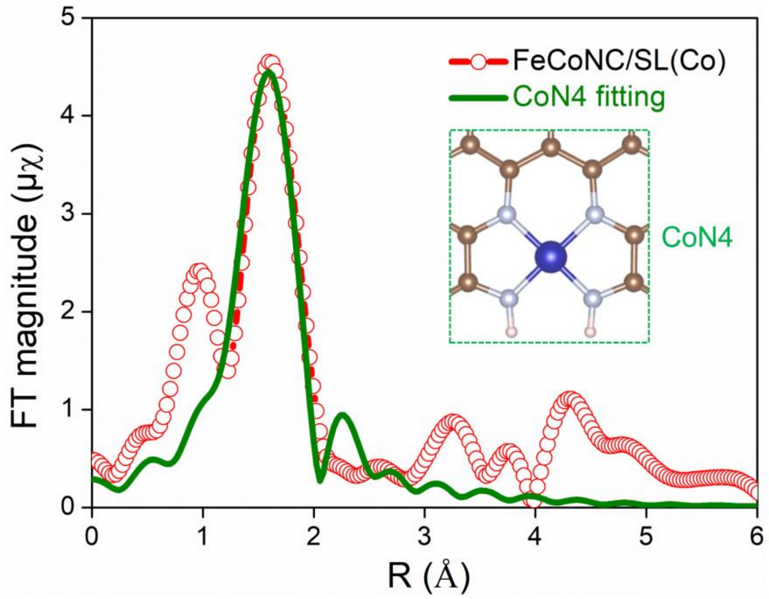


**Figure S15.** XAFS signal obtained by experimental collection (FeCoNC/SL) and theoretical simulation (CoN4C), respectively

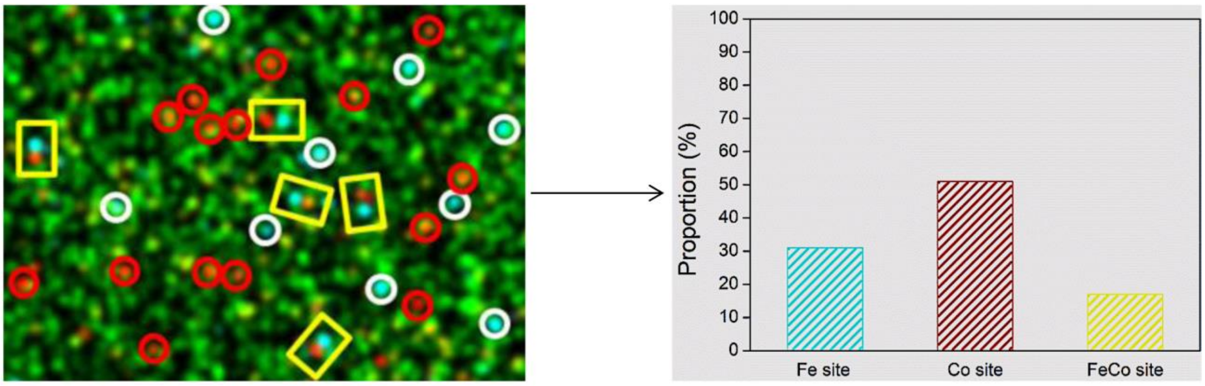


**Figure S16**. Rough statistics of the ratio of various metal sites in FeCoNC/SL based on high-resolution element mapping

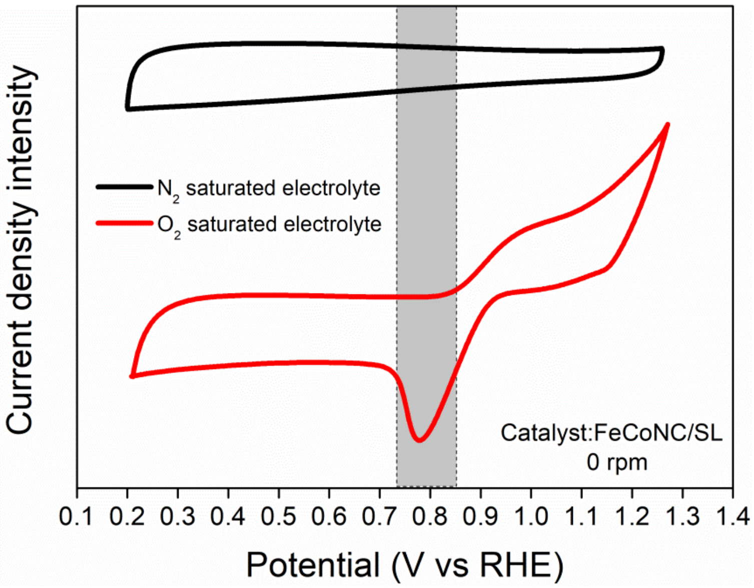


**Figure S17**. CV curves collected after the electrolyte (0.1 M KOH solution) was saturated with $N_2$ and $O_2$ when FeCoNC/SL was used as the catalyst.

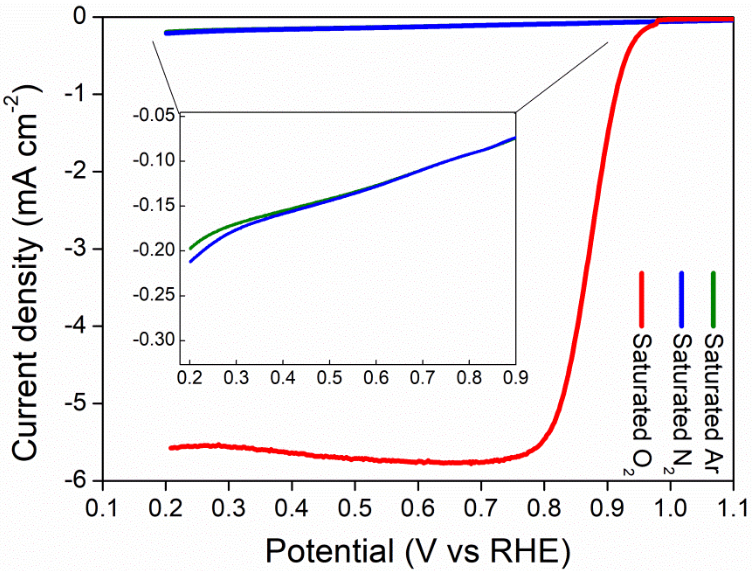


**Figure S18**. When FeCoNC/SL was used as the catalyst, the LSV curves were collected after the electrolyte (0.1 M KOH solution) was saturated with Ar, $N_2$ and $O_2$ respectively, where the rotation rate of the glassy carbon electrode was 1600 rpm.

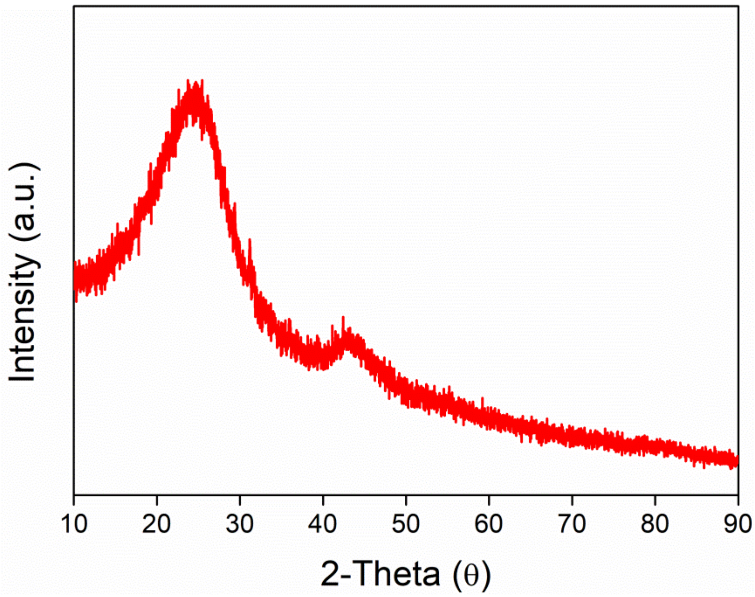


**Figure S19**. PXRD of FeCoNC/SL after use.

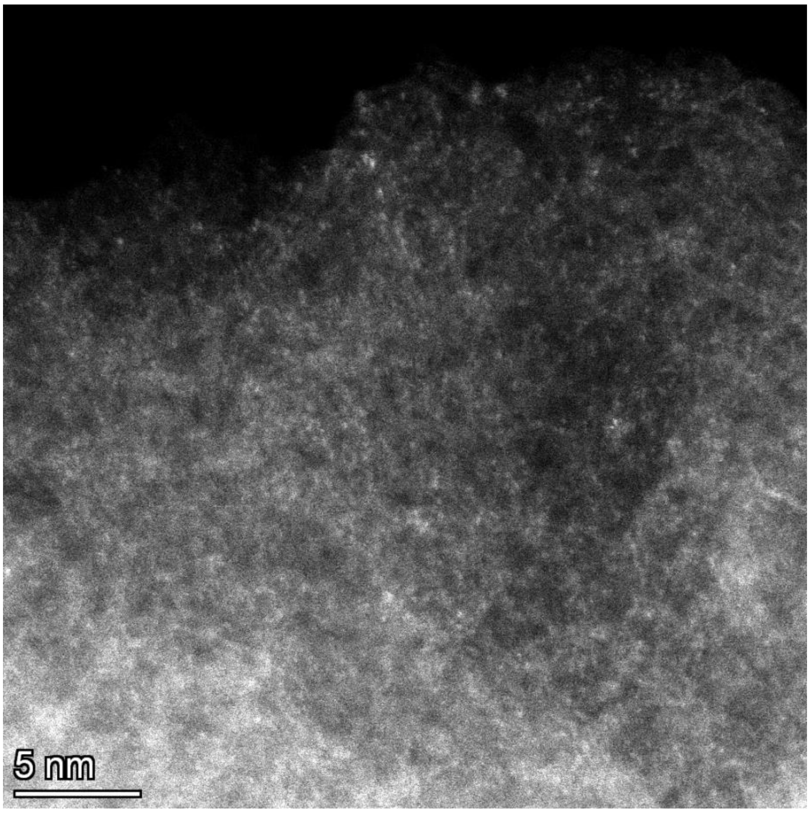


**Figure S20**. HAADF image of FeCoNC/SL after use.

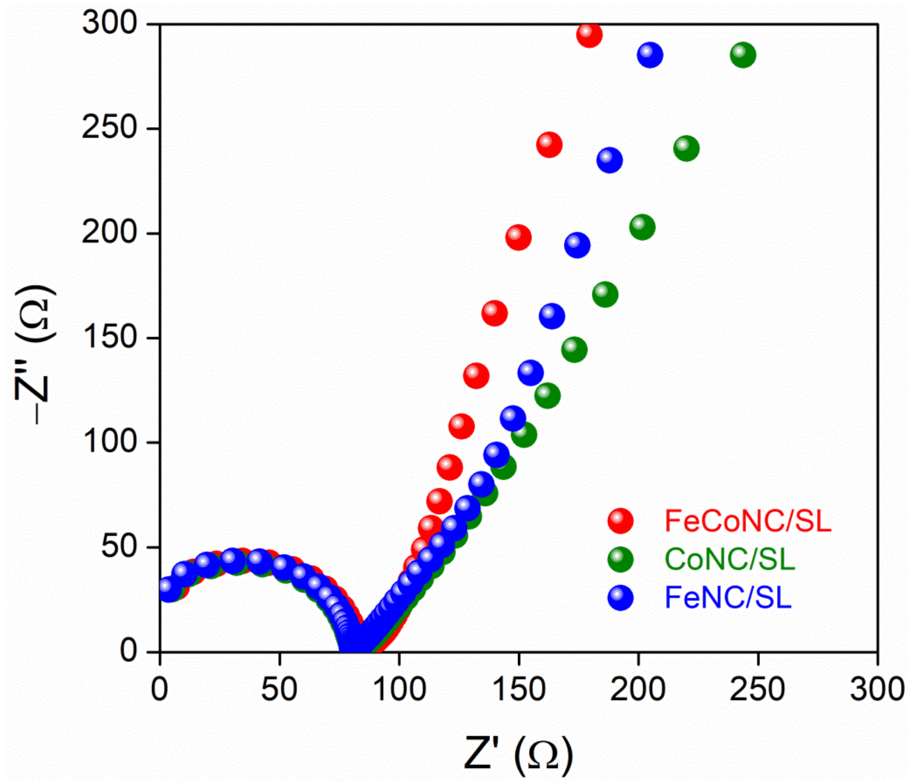


**Figure S21**. Nyquist plots of FeCoNC/SL, FeNC/SL and CoNC/SL

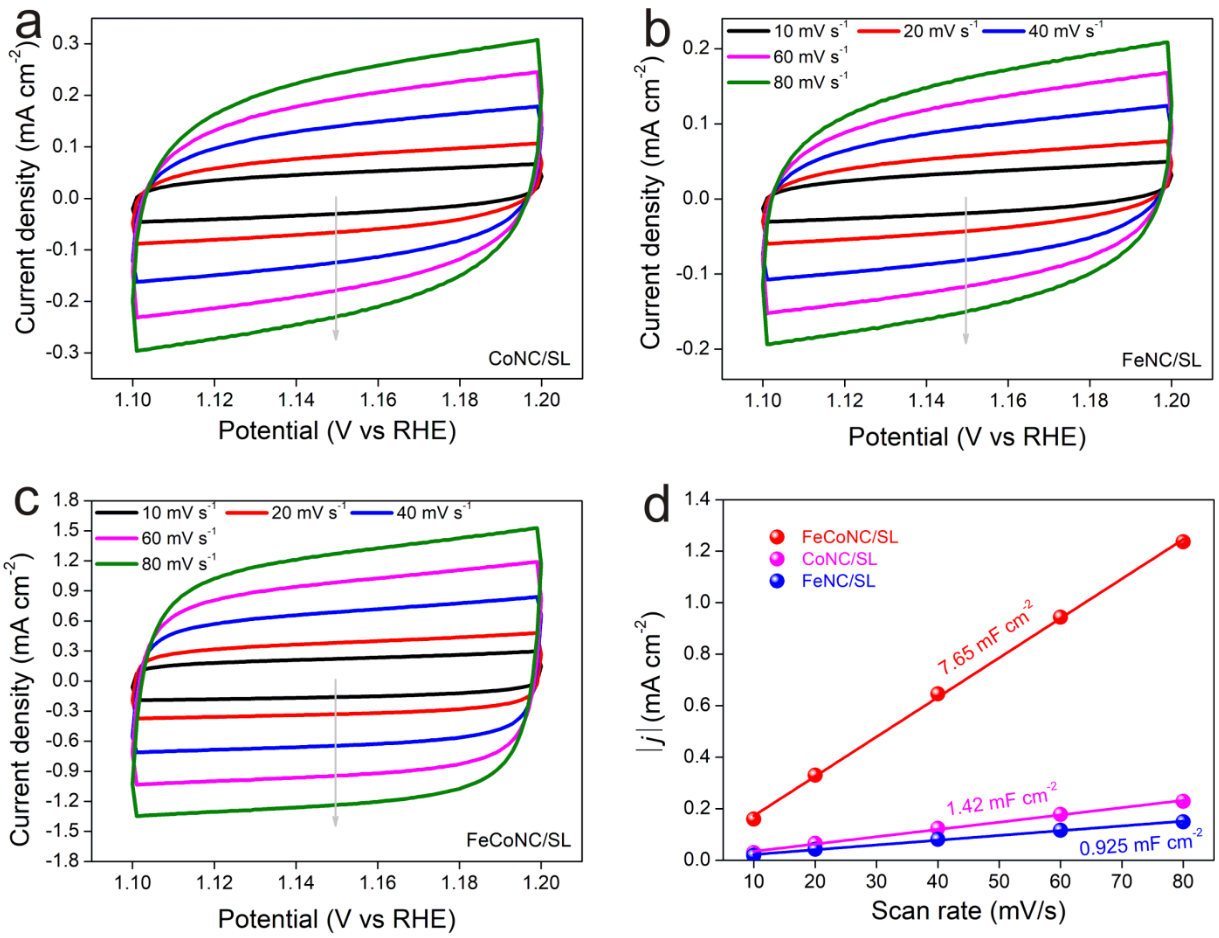


**Figure S22**. (a-c) CV curves of CoNC/SL, FeNC/SL, and FeCoNC/SL in 0.1 M KOH solution; (d) j - scan rate relationship.

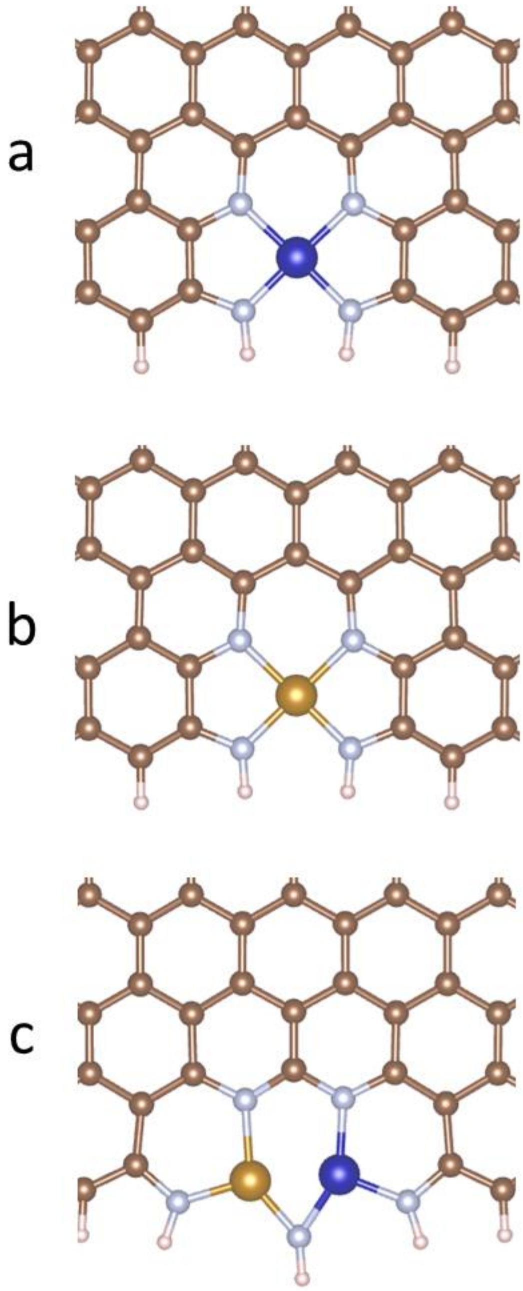


**Figure S23**. Structural models of FeN4C (a), CoN4C (b) and FeCoN5C (c) after energy optimization

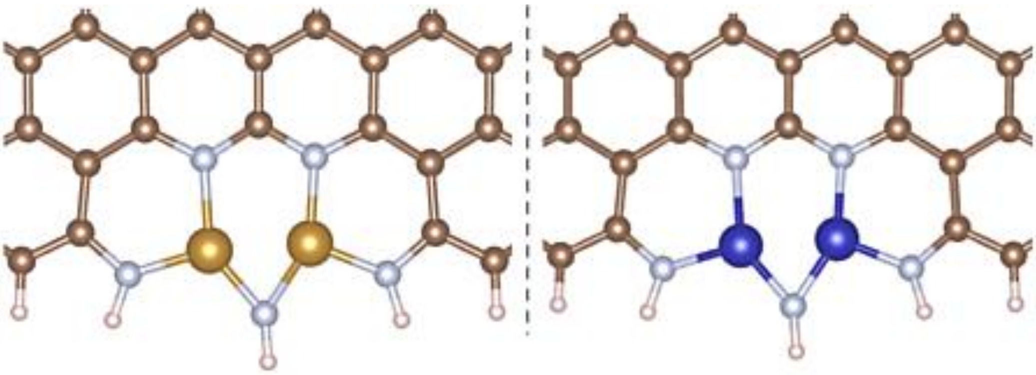

**Figure S24**. Structural models of FeFeN5C (left) and CoCoN5C (right) after energy optimization

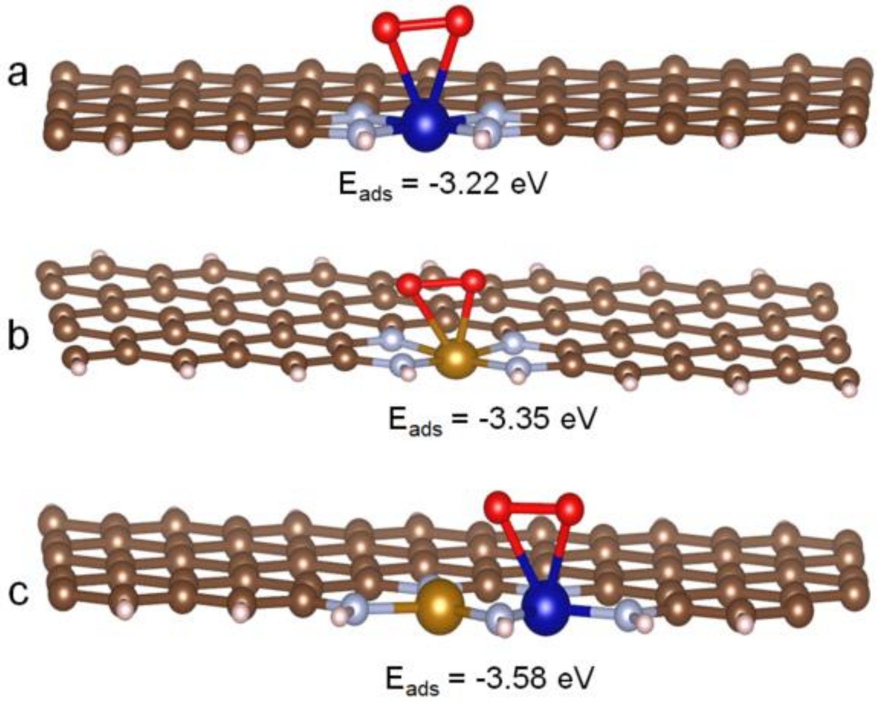


**Figure S25.** The optimized structure of $O_2$ adsorbed on FeN4C (Fe site), CoN4C (Co site), and FeCoN5C (Fe site), respectively. a) FeN4C with $O_2$; b)CoN4C with $O_2$; c) FeCoN5C with $O_2$.

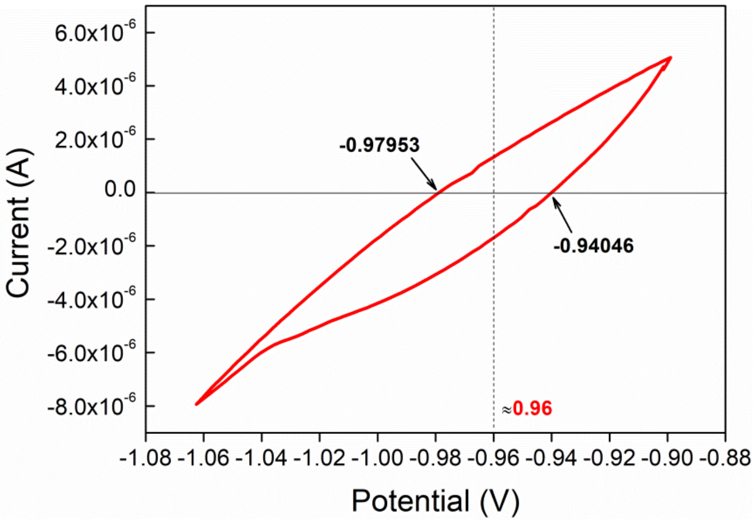


**Figure S26**. Use hydrogen to calibrate the electrode potential of the reference electrode, where the working electrode and the counter electrode are both Pt disks, the reference electrode is a Hg/HgO electrode, and the electrode solution is a 1 M KOH solution saturated with $H_2$.

**Table S1. Summary of EXAFS fitting parameters.**

| Sample | Structure | Bond type | CN[a] | R(Å)[b] | $\sigma^2$ (Å$^2$)[c] | $\Delta E_0$(eV)[d] | R-factor[e] |
|---|---|---|---|---|---|---|---|
| FeCoNC/SL(Co) | CoN4C | Co-N | 4.31 | 1.86 | 0.014 | -8.22 | 0.0212 |
| | FeCoN5C | Co-N | 3.23 | 1.82 | 0.004 | -3.16 | 0.0102 |
| FeCoNC/SL(Fe) | FeN4C | Fe-N | 4.16 | 1.89 | 0.011 | -6.71 | 0.0207 |
| | FeCoN5C | Fe-N | 3.40 | 1.82 | 0.002 | -3.85 | 0.0111 |

Note: $s_0^2$ was fixed as 0.9; $\Delta E_0$ is inner potential correction ($|\Delta E_0|$ should be less than 10 eV); $\sigma^2$ is Debye-Waller factor to account for both thermal and structural disorders; CN is the coordination number; R is interatomic distance (Co-N or Fe-N); R-factor indicates the goodness of the fitting. When XAS data is Co (FeCoNC/SL(Co)), Fourior transform parameters was set as follows: $k_{min}$=3.0, $k_{max}$=10.330, $r_{min}$=1, $r_{max}$=3. When XAS data is Fe (FeCoNC/SL(Fe)), Fourior transform parameters was set as follows: $k_{min}$=3.0, $k_{max}$=10.239, $r_{min}$=1, $r_{max}$=3.

| Table S2. Performance summary of ORR and OER performances of diatomic catalysts. | | | |
|---|---|---|---|
| **Catalysts** | **$E_{1/2}$ of ORR (V vs RHE)** | **$\eta_{10}$ of OER (V)** | **References** |
| **FeCo** | **0.876** | | **This work** |
| FeCo | 0.79 | - | *ChemCatChem*, 2020, 12, 3230–3239. |
| FeCo | 0.86 | - | *Small*, 2020, 16, 2000742. |
| FeCo | 0.86 | - | *Adv. Funct. Mater.*, 2021, 31, 2011289. |
| FeCo | 0.86 | - | *J. Am. Chem. Soc.*, 2019, 141, 17763–17770. |
| FeCo | 0.863 | - | *J. Am. Chem. Soc.*, 2017, 139, 17281–17284 |
| FeCo | 0.89 | - | *Energy Environ. Sci.*, 2019, 12, 1317–1325. |
| FeCo | 0.89 | - | *Adv. Funct. Mater.*, 2021, 31, 2006533. |
| FeCo | 0.87 | 0.44 | *J. Catal.*, 2021, 397, 223–232. |
| FeCo | 0.86 | 0.36 | *Small Methods*, 2020, 5, 2000751. |
| FeCo | 0.877 | 0.349 | *ACS Catal.*, 2020, 12, 1216–1227. |
| FeCo | 0.87 | 0.31 | *Nano Res.*, 2020, 13, 1090–1099. |
| FeNi | 0.79 | - | *J. Phys. Chem. Lett.*, 2020, 11, 1404–1410. |
| FeNi | 0.86 | - | *J. Phys. Chem. Lett.*, 2020, 11, 1404–1410. |
| FeNi | 0.85 | 0.45 | *Nano Energy*, 2020, 71, 104597 |
| FeNi | 0.85 | 0.449 | *Appl. Catal. B*, 2020, 274, 119091. |
| FeNi | 0.83 | 0.41 | *ChemElectroChem*, 2019, 6, 3478–3487. |
| FeNi | 0.83 | 0.39 | *Adv. Mater.*, 2020, 32, 2003134. |
| FeNi | 0.861 | 0.322 | *Appl. Catal.*. B, 2021, 285, 119778. |
| FeNi | 0.84 | 0.27 | *Adv. Energy Mater.*, 2021, 11, 2101242. |
| FeNi | 0.892 | 0.298 | *Energy Storage Mater.*, 2021, 35, 723–730. |
| FeCu | 0.86 | - | *J. Mater. Chem. A*, 2020, 8, 16994–17001. |
| FeZn | 0.873 | - | *Appl. Surf. Sci.*, 2021, 546, 148934. |
| CoZn | 0.797 | - | *J. Mater. Chem. A*, 2020, 8, 3686–3691. |
| CoZn | 0.861 | - | *Angew. Chem. Int. Ed.*, 2019, 58, 2622–2626. |
| CoZn | 0.861 | - | DOI: 10.1002/anie.201810175 |
| CoNi | 0.76 | 0.34 | *Adv. Mater.*, 2019, 31, 1905622. |
| CoNi | 0.880 | - | DOI: 10.1002/adfm.202210867 |

**Table S3**. Performance of aqueous rechargeable ZABs with metal-doped carbon catalysts

| Air catalysts | Electrolytes | Power density ($mW\ cm^{-2}$) | Capacity ($mA\ h\ g_{Zn}^{-1}$) | Ref. |
|---|---|---|---|---|
| Fe–N–C–rich pyrolysis-free COF network | 8 M KOH and 0.5 M ZnO | 123.43 | 732 | [1] |
| Fe-MOF@CNTs–G hybrids | 6 M KOH and 0.2 M $Zn(OAc)_2$ | 95.3 | 637.4 | [2] |
| Fe,N co-doped carbon fibers (FeNCF) | 6 M KOH and 0.2 M $Zn(OAc)_2$ | 145 | 784.3 | [3] |
| FeS/Fe3C@NS-C-900 | 6 M KOH and 0.2 M $Zn(OAc)_2$ | 90.9 | 750 | [4] |
| Binary NixFe100x-NC (x = 0–100) materials | 6 M KOH and 0.2 M $Zn(OAc)_2$ | 140.1 | 765.5 | [5] |
| Fe-N-C/N-OMC | 6 M KOH and 0.2 M $Zn(OAc)_2$ | 113 | 711 | [6] |
| Single-atom Fe-Nx-C | 6 M KOH and 0.2 M $Zn(OAc)_2$ | 96.4 | 641 | [7] |
| **FeCoNC/SL** | 6 M KOH and 0.2 M $Zn(OAc)_2$ | **224.8** | **803** | **This Work** |

**Table S4. Formation energy of different structural models.**

| Structure | Formation energy of model |
|---|---|
| FeN4C | -6.334 eV |
| CoN4C | -6.517 eV |
| FeFeN5C | -7.254 eV |
| CoCoN5C | -7,428 eV |
| FeCoN5C | -7.085 eV |

**Table S5.** The loading mass ratio of the metal in the sample.

| Sample | Co wt% | Fe wt% |
|---|---|---|
| FeNC/SL | - | 0.79 |
| CoNC/SL | 1.01 | - |
| FeCoNC/L | 0.53 | 0.41 |

[1] P. Peng, L. Shi, F. Huo, C. Mi, X. Wu, S. Zhang, Z. Xiang, Sci. Adv. 2019, 5, eaaw2322.
[2] W. Yang, Y. Zhang, X. Liu, L. Chen, J. Jia, Chem. Commun. 2017, 53, 12934.
[3] J. Zhang, Y. Liu, Z. Yu, M. Huang, C. Wu, C. Jin, L. Guan, J. Mater. Chem. A 2019, 7, 23182.
[4] Y.-W. Li, W.-J. Zhang, J. Li, H.-Y. Ma, H.-M. Du, D.-C. Li, S.-N. Wang, J.-S. Zhao, J.-M. Dou, L. Xu, ACS Appl. Mater. Inter. 2020, 12, 44710.
[5] M. Ma, A. Kumar, D. Wang, Y. Wang, Y. Jia, Y. Zhang, G. Zhang, Z. Yan, X. Sun, Appl. Catal., B 2020, 274, 119091.
[6] J. Han, H. Bao, J.-Q. Wang, L. Zheng, S. Sun, Z. L. Wang, C. Sun, Appl. Catal., B 2021, 280, 119411.
[7] J. Han, X. Meng, L. Lu, J. Bian, Z. Li, C. Sun, Adv. Funct. Mater. 2019, 29, 1808872.
